# Quasi-square-shaped cadmium hydroxide nanocatalysts for electrochemical CO_2_ reduction with high efficiency†

**DOI:** 10.1039/d1sc02328d

**Published:** 2021-08-10

**Authors:** Chunjun Chen, Xupeng Yan, Ruizhi Wu, Yahui Wu, Qinggong Zhu, Minqiang Hou, Zhaofu Zhang, Honglei Fan, Jun Ma, Yuying Huang, Jingyuan Ma, Xiaofu Sun, Longfei Lin, Shoujie Liu, Buxing Han

**Affiliations:** Beijing National Laboratory for Molecular Sciences, CAS Key Laboratory of Colloid and Interface and Thermodynamics, CAS Research/Education Center for Excellence in Molecular Sciences, Institute of Chemistry, Chinese Academy of Sciences Beijing 100190 P. R. China hanbx@iccas.ac.cn sunxiaofu@iccas.ac.cn linlongfei@iccas.ac.cn; University of Chinese Academy of Sciences Beijing 100049 China; Chemistry and Chemical Engineering of Guangdong Laboratory Shantou 515063 China; College of Chemistry and Materials Science, Anhui Normal University Wuhu 241000 China jiesliu@ahnu.edu.cn; Shanghai Synchrotron Radiation Facility, Zhangjiang Laboratory (SSRF, ZJLab), Shanghai Advanced Research Institute, Chinese Academy of Sciences Shanghai 201204 China; Shanghai Key Laboratory of Green Chemistry and Chemical Processes, School of Chemistry and Molecular Engineering, East China Normal University Shanghai 200062 China

## Abstract

Powered by a renewable electricity source, electrochemical CO_2_ reduction reaction is a promising solution to facilitate the carbon balance. However, it is still a challenge to achieve a desired product with commercial current density and high efficiency. Herein we designed quasi-square-shaped cadmium hydroxide nanocatalysts for CO_2_ electroreduction to CO. It was discovered that the catalyst is very active and selective for the reaction. The current density could be as high as 200 mA cm^−2^ with a nearly 100% selectivity in a commonly used H-type cell using the ionic liquid-based electrolyte. In addition, the faradaic efficiency of CO could reach 90% at a very low overpotential of 100 mV. Density functional theory studies and control experiments reveal that the outstanding performance of the catalyst was attributed to its unique structure. It not only provides low Cd–O coordination, but also exposes high activity (002) facet, which requires lower energy for the formation of CO. Besides, the high concentration of CO can be achieved from the low concentration CO_2_*via* an adsorption-electrolysis device.

## Abstract 2

Quasi-square cadmium hydroxide nanocrystals (Cdhy-QS) showed outstanding performance for electroreduction CO_2_ to CO.

## Graphical abstract

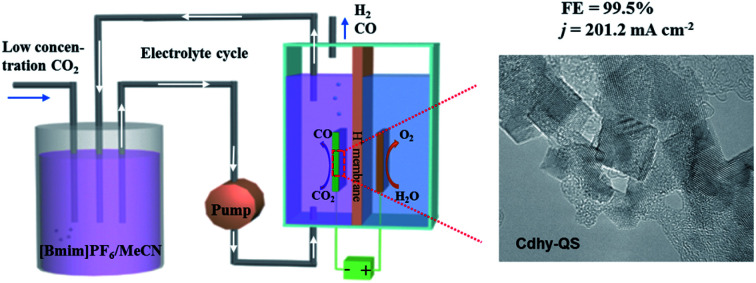

## Introduction

The concentration of atmospheric carbon dioxide (CO_2_) increased dramatically with the consumption of fossil fuels, which has caused climate change and serious environmental issues.^1–5^ Electrochemical CO_2_ reduction reaction (CO_2_RR) is a promising solution to facilitate the carbon balance, which can not only transfer CO_2_ into valuable fuels and chemicals, but also provide a solution for storage of the renewable energy.^6–10^ CO is a versatile feedstock for producing various chemicals and fuels, which is viewed as the closest to commercialization among all the products.^11–16^ Although the faradaic efficiency (FE) and current density for CO have been significantly improved by using some noble-based metals (Pd, Au, Ag, *etc.*),^17–19^ non-noble based metals (Fe, Co, Ni, *etc.*)^20–22^ and metal-free carbon materials.^23–26^ More efficient electrocatalysts that meet the commercial purpose still remain to be developed.

Recently, Cd-based materials (*e.g.*, CdS, CdSe and metallic Cd) have been reported to convert CO_2_ to CO efficiently, due to the Cd sites can suppress the hydrogen evolution reaction (HER) and exhibit excellent CO anti-poisoning.^27–29^ The previous works showed that tuning the coverage of surface hydroxyl groups and developing special morphology of catalysts can promote CO_2_ reduction and inhibit the HER.^30,31^ As an important and conventional semiconductor, cadmium hydroxide (Cdhy) has been applied in a wide range of fields, including solar cells, sensors, and cathode electrode materials of batteries.^32,33^ It offers an opportunity for developing novel Cdhy catalysts, which may be favorable for improving the selectivity and activity of CO from CO_2_RR.

In addition, using the industrial outputs as the CO_2_ source is commercially viable. The concentration of CO_2_ in flue gas in the industrial processes (*i.e.* steel and cement production, power plant flue gas) is usually between 4% and 15%.^34^ To achieve more commercial feasibility of CO_2_RR, the CO_2_ reactant and the products separation should be considered simultaneously. Although the selectivity and activity of CO_2_RR have been significantly improved in the flow cell and membrane electrode assembly (MEA),^35,36^ the CO_2_ availability is low, and the product separation is also high energy consumption. In addition, the alkaline electrolyte was used, which can result in the CO_2_ loss, imposing a limit of carbon utilization efficiency.^37^ According to the previous reports,^38^ the low concentrations of CO_2_ can be directly converted to CO, however, the concentration of the CO was very low. Recently, high content of CO has been obtained *via* the electroreduction of the captured solution of CO_2_.^39^ However, the FE and current density were only 70% and 50 mA cm^−2^, which was limited to the poor electrocatalytic performance of capture solution. In our previous reports,^40–42^ the ionic liquid (IL) has been proven to be a good solvent for absorbing CO_2_, and the IL was also an excellent electrolyte for CO_2_RR. Thus, we can assume that combining the feature of trapping and electrocatalytic activity of IL for CO_2_ can promote the development of commercialization.

Herein we developed a feasible and facile strategy to synthesize quasi-square-shaped Cdhy-QS nanocatalysts for ultra-efficient CO_2_ electroreduction. Cdhy-QS yielded nearly 100% CO selectivity in a very wide potential range with extremely high current density (>200 mA cm^−2^). Moreover, the energy efficiency (EE) was also very high. These advantages made Cdhy-QS have great potential for practical application. X-ray absorption spectroscopy (XAS) and density functional theory (DFT) calculations showed that high activity facet (002) exposed on the surface and the low Cd–O coordination number resulted in the enhancement of CO_2_ activation. In addition, the high concentrations of CO in the gas products can be achieved from the low concentrations of CO_2_, when combining the Cdhy-QS and the adsorption-electrolysis device system.

## Results and discussion

**Fig. 1:**
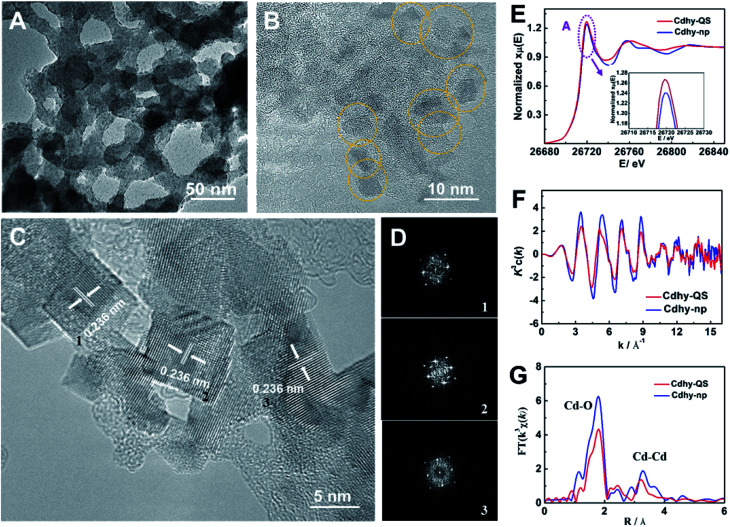
(A and B) TEM images of Cdhy-QS. (C) HR-TEM image of Cdhy-QS. (D) The FFT pattern of the corresponding HR-TEM. (E) XANES spectra at the Cd K-edge. (F) Cd K-edge extended XAFS oscillation function *k*^3^c(*k*). (G) The corresponding Fourier transforms FT(*k*^3^*χ*(*k*)).

We used tannic acid (TA) as capping agent, which is commonly used to control synthesis of functional materials,^38^ to synthesize Cdhy-QS (see the experimental details in the ESI†). Firstly, X-ray diffraction (XRD) analysis was used to characterize Cdhy-QS, and the features of Cd(OH)_2_ can be observed (Fig. S1†). From transmission electron microscope (TEM) image of Cdhy-QS (Fig. 1A), we can observe that the Cdhy was dispersed homogeneously on the cross-linked architecture of TA. And the size of Cdhy-QS was uniform (Fig. 1B) with a quasi-square shape, which has not been reported in the literature for Cdhy. The energy dispersive X-ray elemental (EDX) mappings show that the Cd and O elements were homogenous over the entire architectures (Fig. S2†). The high-resolution TEM images reveals that Cdhy-QS had an interplanar spacing of 0.236 nm (Fig. 1C), which corresponds to the (002) plane of Cdhy. It was also identified by the corresponding fast Fourier transform (FFT) pattern (Fig. 1D). They are different with those of Cdhy nanoparticles (Cdhy-np) (see the experimental details in the ESI†), which are (100) and (110) facets (Fig. S3†). The content of Cd in Cdhy-QS and Cdhy-np was 9.5 wt% and 9.8 wt%, respectively, which were determined by inductively coupled plasma-atomic emission spectrometry (ICP-AES). Additionally, enlarged and repeated experiments for Cdhy-QS preparation were carried out (Fig. S4 and S5†), and 16 g Cdhy-QS was obtained in one experiment that only took 30 minutes, and the morphologies were similar.

The local coordination environment of Cdhy-QS and Cdhy-np were characterized by synchrotron radiation X-ray absorption fine structure spectroscopy (XAFS) measurements. From the normalized Cd K-edge X-ray absorption near edge structure (XANES), we can observe that the post-edge curve for Cdhy-QS exhibited obvious differences in comparison with Cdhy-np (Fig. 1E). The white line of Cd K-edge (peak A) of Cdhy-QS is higher than that of Cdhy-np, which is corresponding to a 1s–4p transition and suggests that the electronic properties of Cd atom in Cdhy-QS are modified *via* changing the coordination of Cd–O. However, the threshold value of Cd K-edge for Cdhy-QS and Cdhy-np have the same spectral shape, indicating that Cdhy-QS and Cdhy-np have similar valence state. The oscillation *k*^3^*χ*(*k*) functions (Fig. 1F) showed that the *k*^3^*χ*(*k*) of Cdhy-QS was obvious different from that of Cdhy-np in all range of *k* region. The low *k* region (*k* = 3–8 Å^−1^) and high *k* region (*k* = 9–13 Å^−1^) can be ascribed to the Cd–O and Cd–Cd coordination, respectively. It can be seen that the amplitude of *k*^3^*χ*(*k*) for Cdhy-QS was lower than that of Cdhy-np, especially in the low *k* region. It suggests that the decrease of Cd–O coordination number was more significant than that of Cd–Cd. Such changes in the coordination number of Cd–O and Cd–Cd were further showed in the Fourier-transform (FT) of EXAFS in Fig. 1G. The FT curves of Cdhy showed an obvious Cd–O peak located at 1.8 Å^−1^, as well as Cd–Cd peak located in the range of 2.6–3.8 Å^−1^. For Cdhy-QS, the intensity of Cd–O coordination decreased more significantly than that of Cd–Cd coordination, indicating Cdhy-QS had less O coordination. Moreover, we used the ARTEMIS programs of IFEFFIT to fit the Cd–O and Cd–Cd coordination number for the as-prepared materials. The results are provided in Fig. S6 and Table S1.† For Cdhy-QS and Cdhy-np, the Cd–O coordination number (N_Cd–O_) were 4.0 and 5.7, and Cd–Cd coordination number (N_Cd–Cd_) were 1.8 and 2.2, respectively. The ratios of N_Cd–O_/N_Cd–Cd_ for Cdhy-QS and Cdhy-np were 2.22 and 2.59, respectively, indicating that Cdhy-QS exhibited more uncoordinated Cd–O bond than Cdhy-np. Therefore, the fine structure of Cdhy-QS was distinguished from that of Cdhy-np.

**Fig. 2:**
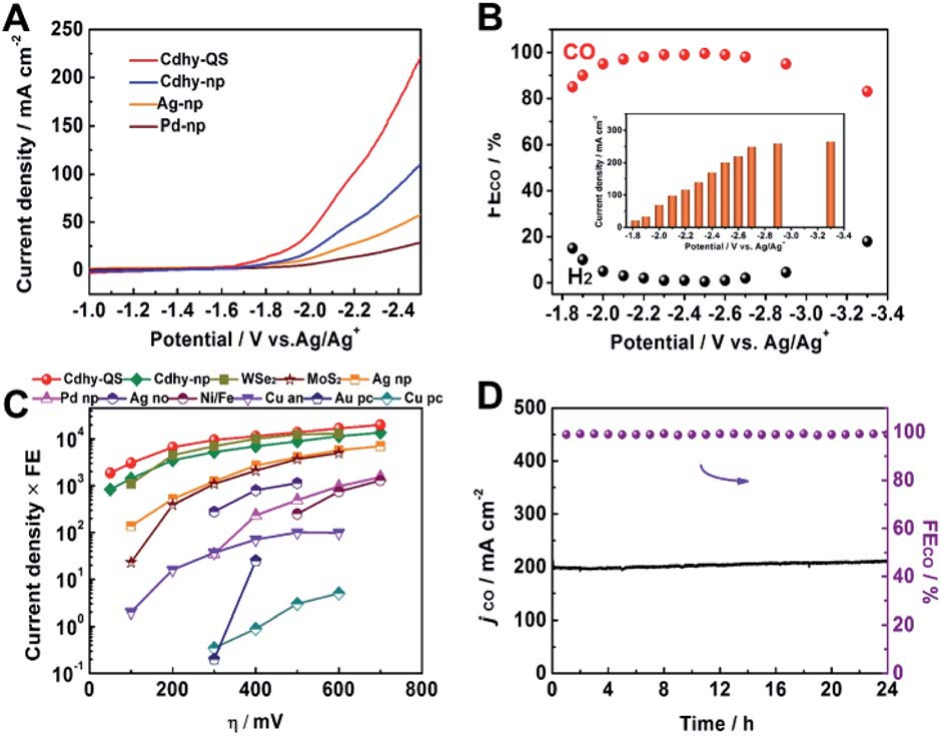
(A) LSV of different catalysts in CO_2_-saturated 0.5 M [Bmim]PF_6_/MeCN electrolyte. (B) The FE for CO and current density over Cdhy-QS in 0.5 M [Bmim]PF_6_/MeCN electrolyte at different potentials. (C) The dependence of current density time FE on overpotential (*η*) for different catalysts. The data of Cdhy-QS, Cdhy-np, Ag-np and Pd-np were obtained from chronoamperometry experiments under identical conditions. The detail data for other catalysts are listed in Table S2.† (D) Long-term stability of Cdhy-QS at −2.5 *vs.* Ag/Ag^+^ for 24 h.

To characterize the electrocatalytic activity for CO_2_ reduction, the as-prepared catalysts were dispersed in acetone with carbon black and poly(tetrafluoroethylene) to form a homogeneous ink. They were spread and pressed on a nickel foam substrate under 4 MPa, which served as the working electrode (Fig. S7†). Imidazolium-based ionic liquid (IL)/MeCN solution was used as the electrolyte due to the significant advantages of IL, including enhancing CO_2_ solubility, reducing the overpotential and suppressing the HER.^43,44^ The linear sweep voltammetry (LSV) was first carried out in CO_2_-saturated 0.5 M [Bmim]PF_6_/MeCN solution using a standard three-electrode configuration. Cdhy-QS showed higher current density than that of Cdhy-np (Fig. 2A). It was also significantly higher than that over Ag nanoparticles (Ag-np) and Pd nanoparticles (Pd-np), which were usually considered as efficient catalysts for CO_2_ electroreduction to CO. Meanwhile, much lower current density was observed in N_2_-saturated environment (Fig. S8†), indicating the occurrence of CO_2_ reduction under the applied potentials.

Controlled potential electrolysis of CO_2_ was performed in a typical H-type cell separated by a Nafion-117 membrane at various potentials. The gaseous and liquid products were then analyzed by gas chromatography (GC) and nuclear magnetic resonance (NMR) spectroscopy, respectively. The dominant product was CO when the [Bmim]PF_6_/MeCN solution was used as the electrolyte. However, only a small amount of CO was detected when the electrolysis was performed in KHCO_3_ or KOH aqueous solution. These results indicated that the [Bmim]PF_6_/MeCN solution exhibited unique advantage for CO_2_ electroreduction. To confirm the carbon source, the blank experiment was performed by using N_2_ to replace CO_2_ during the electrolysis, and no CO was detected. The isotope-labeled experiment was also performed by using ^13^CO_2_ to replace CO_2_, and only ^13^CO signal can be found from GC-mass spectrometry (GC-MS) spectra (Fig. S9†). These data confirmed that CO_2_ was the only carbon source.

It can be clearly observed that Cdhy-QS showed outstanding activity for CO_2_ electroreduction. The FE_CO_ was nearly 100% from −2.3 to −2.6 V *vs.* Ag/Ag^+^ (Fig. 2B). Even at a very low overpotential of 50 mV (−1.85 V *vs.* Ag/Ag^+^, Fig. S10†), the FE_CO_ can still reach 85.0%. For comparison, we also investigated the CO_2_ activation for Cdhy-np, Ag-np and Pd-np under the same reaction conditions (Fig. S11–S13†). The FE of Cdhy-np for CO was only 76.1%, while Ag-np and Pd-np could not reduce CO_2_ at such low potential. In the meantime, we also calculated the EE for the conversion of CO_2_ into CO. It was found that the EE_CO_ exceeded 70% at a wide potential range of −1.85 to −2.7 V *vs.* Ag/Ag^+^ for Cdhy-QS (Fig. S14†). Such high FE_CO_ and EE_CO_ in a wide range potential is unusual.

The Cdhy-QS exhibited a very high current density for CO production at all applied potentials (Fig. S15†). The current density for CO production was 18.7 mA cm^−2^ (normalized by geometrical surface area) at an overpotential of 50 mV, which was roughly 2.6 times higher than that of Cdhy-np. More importantly, it could reach a high value of 201.2 mA cm^−2^ with FE of 99.5% at −2.5 V *vs.* Ag/Ag^+^, which is outstanding in both current density and FE comparing with the data reported in the literature (Table S2†).

The catalytic activity of Cdhy-QS was compared with other reported catalysts (Fig. 2C) by multiplying current density (activity) by CO formation FE (selectivity). At an overpotential of 100 mV, the performance of Cdhy-QS exceeded that of Cdhy-np and Ag-np by a factor of about 2.1 and 22, respectively. It exceeded that of WSe_2_ (ref. 43) and MoS_2_ (ref. 45) tested under similar conditions by a factor of nearly 2.8 and 133, respectively. Additionally, the performance of Cdhy-QS was also much better than that of other commonly used metals, such as Au^46^ and Cu,^47^ by three orders of magnitude. These results indicated that Cdhy-QS had outstanding performance for CO_2_ reduction.

Furthermore, continuous CO_2_ reduction was performed at −2.5 V *vs.* Ag/Ag^+^ for 24 h to elucidate the electrode stability. As shown in Fig. 2D, there was no decay in both current density and FE for CO. We also characterized Cdhy-QS after 24 h electrolysis *via* TEM, X-ray photoelectron spectroscopy (XPS) and LSV (Fig. S16†). No obvious changes in morphology, structure and electrochemical characteristics were observed, further showing the excellent stability of Cdhy-QS in the reaction.

**Fig. 3:**
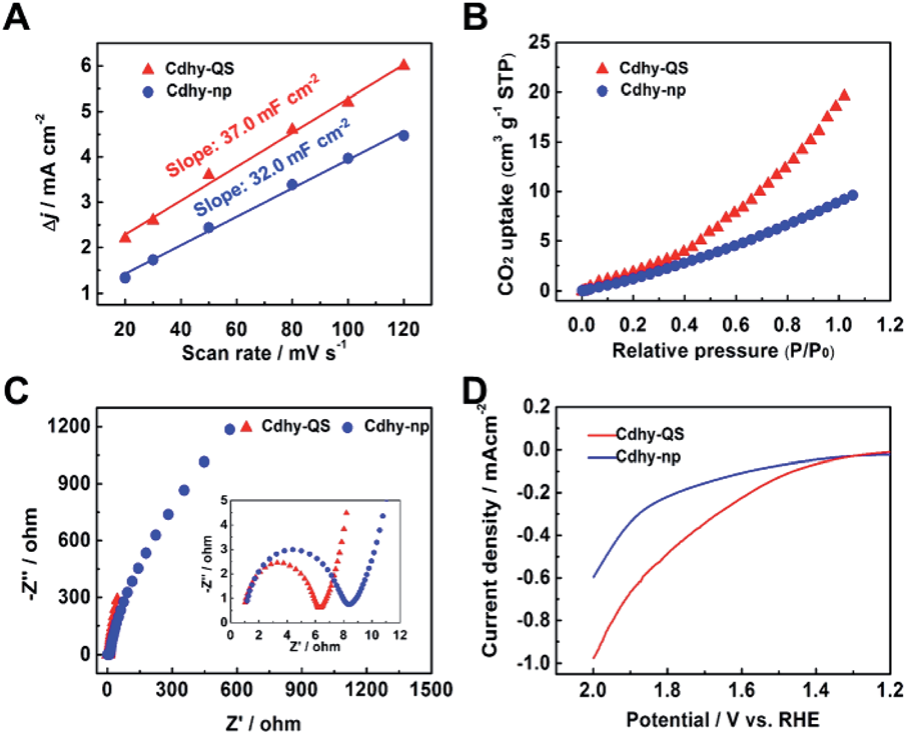
(A) Charging current density different Δ*j* plotted against scan rates. (B) The CO_2_ adsorption behaviors of Cdhy-QS and Cdhy-np. (C) Nyquist plots for Cdhy-QS and Cdhy-np in CO_2_-saturated 0.5 M [Bmim]PF_6_/MeCN electrolyte. (D) Single oxidative LSV curves for SO_4_^2−^ adsorption in N_2_-saturated 0.1 M Na_2_SO_4_.

The intrinsic reason for the excellent catalytic performance of Cdhy-QS was further investigated. According to cyclic voltammograms (CV) curves under different scan rates, the electrochemical active surface areas (ECSAs) of Cdhy-QS and Cdhy-np were determined by measuring double layer capacitance (*C*_dl_; Table S3†). The ECSAs of Cdhy-QS was slightly higher than that of Cdhy-np (Fig. 3A), suggesting that Cdhy-QS had larger amount of active sites. The total current density and partial current density for CO production were further normalized by ECSAs (Fig. S17†). Similar to the geometric current density, both of them over Cdhy-QS were significantly higher than those of Cdhy-np. Therefore, the higher activity of Cdhy-QS was mainly attributed to its special structure and morphology, rather than increasing number of active sites. Moreover, Cdhy-QS had higher CO_2_ adsorption capacity, verified by the CO_2_ adsorption isotherms in Fig. 3B. It could reach 19.6 cm^3^ g^−1^ at 1 atm, which was roughly 2.2 times higher than that of Cdhy-np. The measured Nyquist plots at an open circuit potential showed that the charge transfer resistance (*R*_ct_) between Cdhy-QS and electrolyte was smaller than that between Cdhy-np and electrolyte (Fig. 3C). This indicated more facile electron transfer from electrolyte to Cdhy-QS electrode surface, resulting in a higher overall electronic conductivity. Additionally, we also examined SO_4_^2−^ adsorption as a surrogate for CO_2_˙^−^ by measuring single oxidative LSV scans between 1.2 V and 2.0 V (*vs.* RHE) at 10 mV s^−1^ in N_2_-saturated 0.1 M Na_2_SO_4_ electrolyte.^48,49^ The overpotential for surface SO_4_^2−^ adsorption on Cdhy-QS was lower than that of Cdhy-np (Fig. 3D), indicating Cdhy-QS could stabilize CO_2_˙^−^ intermediates more efficiently.

**Fig. 4:**
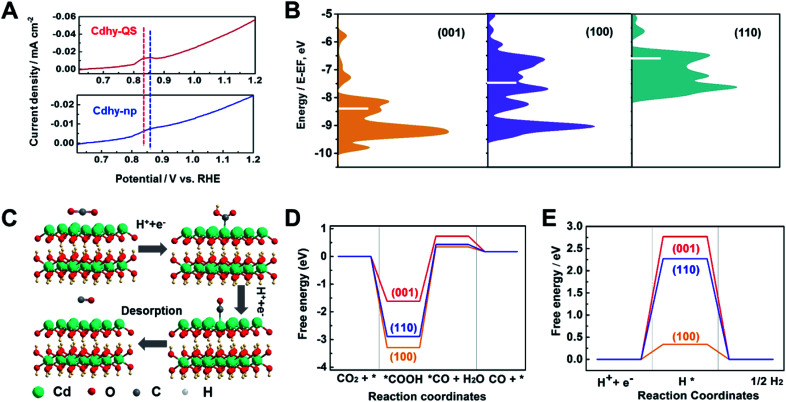
(A) The LSV curves for CO desorption from Cdhy-QS and Cdhy-np in 0.1 M Na_2_SO_4_. (B) The d-band center of gravity for different facets of Cdhy. (C) Proposed reaction paths for CO_2_ reduction to CO over Cdhy-QS. (D) Gibbs free energy diagrams for CO_2_ reduction to CO over different facets of Cdhy. (E) Gibbs free energy diagrams for HER over different facets of Cdhy.

Furthermore, the valence band spectra were determined to further clarify the charge-transfer process for CO_2_ adsorption and activation over Cdhy-QS in electrolysis. As shown in Fig. S18,† the significant change in the valence band can be observed after CO_2_ reduction over Cdhy-QS, which mainly results from the formation and adsorption of CO_2_˙^−^ species.^5^ However, there is no obvious change in the valence band over Cdhy-np. These indicate that Cdhy-QS possessed higher ability for CO_2_ activation and CO_2_˙^−^ intermediates stabilization. The desorption ability of CO is also an important factor affecting the activity of CO_2_ reduction to CO, and it could be probed by the electrochemical CO stripping voltammetry method.^50^ As shown in Fig. 4A, a sharp CO stripping profile with a dominant peak around 0.83 V *vs.* RHE occurred on Cdhy-QS, whereas there is a broad peak around 0.86 V *vs.* RHE on Cdhy-np. The negative shift indicated that Cdhy-QS had stronger CO desorption ability, leading to higher activity for CO_2_ reduction to CO.

DFT calculations were then performed to gain insight into the outstanding performance of Cdhy-QS. Based on the HR-TEM images, the Cdhy-QS exposed (002) facet. In DFT calculations, the (002) facet can be represented by the basic (001) facet, because they are parallel to each other and exhibited the same atomic configuration. Thus the (001) facet can stand for Cdhy-QS, and the (110) and (100) facets represent Cdhy-np. We first optimized the three basic facets of Cdhy (Fig. S19†). For transition metal catalysts, the way that their d-band interacts with the intermediates usually determines the binding strength and the products.^13^ As shown in Fig. 4B, we can observe that the d-band center of (001) facet is more negative than that of other facets. It indicates that the binding strength for *COOH and *CO is weak on (001) facet, which contributes to CO production.^51^ Therefore, (001) facet can be considered as high activity facet of Cdhy.

In addition, the Gibbs free energy diagrams of CO_2_ → CO pathway were also studied (Fig. 4C, D, S20–S22 and Table S4†), *COOH formation on Cdhy surfaces is exergonic because of strong binding to the corresponding sites. The energetic ordering suggests that *COOH is less stable on (001) facet than on (110) and (100) facet, resulting in more favorable for further conversion of *COOH. The formation of *CO + H_2_O from *COOH is highly endergonic and acts as the rate-determining step (RDS). According to the simulation, the free energy for the RDS on (001), (110) and (100) facet are 2.30 eV, 3.32 eV and 3.64 eV, respectively. It is also kinetically favorable on (001) facet compared to (110) and (100) facet, demonstrating that the cycle of CO_2_ to CO is easier on (001) facet than that on (110) and (100) facet. Furthermore, the Gibbs free energy of HER was also calculated (Fig. 4E and S23†), which is a dominant competitive reaction upon CO_2_ reduction. It can be seen that the HER on Cdhy is endergonic. The (001) facet exhibits the highest energy barrier (2.77 eV), suggesting that it can significantly suppress the adsorption of *H. Therefore, the (001) facet can reduce the Gibbs free energy of RDS for CO_2_-to-CO conversion and hinder the HER simultaneously. As a result, Cdhy-QS had better performance for CO production compared with Cdhy-np.

**Fig. 5:**
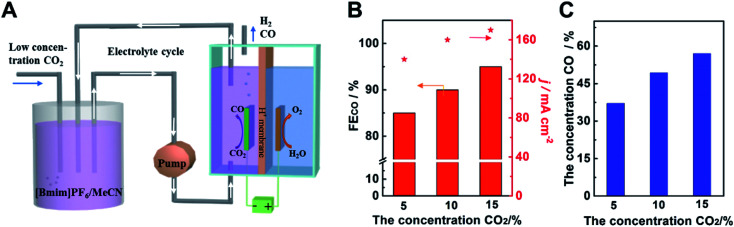
(A) The schematic diagram for the CO_2_ adsorption-electrolysis device system. (B) The FECO and current density for Cdhy-nc using low concentration CO_2_. (C) The concentration of CO in the gas products using low concentration CO_2_.

In order to use the low concentration of CO_2_ and obtain high concentration of CO simultaneously, we designed an adsorption-electrolysis device. The adsorption of CO_2_ and the CO_2_ electroreduction were divided into two parts. As shown in Fig. 5A, the low concentration CO_2_ was bubbled into the adsorption cell first, and CO_2_ can be dissolved in 0.5 M [Bmim]PF_6_/MeCN solution, due to its high adsorption capacity of CO_2_. Different ratios of CO_2_/N_2_ gas were mixed to simulate the exhaust gas. Then the solution was pumped into the electrolytic cell. CO_2_ can be reduced to CO efficiently and high concentration CO products were obtained. After the reaction, the electrolyte can be transferred back to the adsorption cell, achieving the reuse of the electrolyte. We can see that the electroreduction of low concentration CO_2_ was still high-efficiency over Cdhy-QS (Fig. 5B). The FE of CO can reach 84.6%, 89.5% and 94.4%, when the concentration of CO_2_ was 4.9%, 10.1% and 14.8%, respectively. In addition, the concentrations of CO in the gas products were 37.1%, 49.3% and 57.0%, respectively (Fig. 5C and Table S5†). It indicated the catalyst Cdhy-QS is still high activity and the high concentration CO can be obtained when using low concentration CO_2_. Thus we can believe that combining the active catalysts and the adsorption-electrolysis device system will greatly promote the commercialization process of CO_2_RR.

## Conclusions

In summary, we have demonstrated that Cdhy-QS has highly reactive facet on the surface with low Cd–O coordination number, resulting in very high efficiency for CO_2_ electroreduction to CO. The Cdhy-QS catalyst yields nearly 100% CO selectivity in a wide potential range and the current density can be higher than 200 mA cm^−2^ with high energy efficiency. The detailed study indicated that the outstanding catalytic performance of Cdhy-QS can be attributed to the enhanced CO_2_ adsorption and activation, as well as the low charge transfer resistance. We believe that the highly efficient Cdhy-QS catalyst has great potential for practical application. In addition, combining the Cdhy-QS and the adsorption-electrolysis device system can obtain high concentration of CO at low CO_2_ concentration. The findings of this work expand knowledge for the design of novel and efficient electrocatalysts, and also provide a guideline for promoting commercialization of CO_2_RR.

## Data availability

The data that support the findings of this study are available within the article and its ESI.†

## Author contributions

C. J. C., X. F.·S., L. F. L., S. J. L. and B. X. H. proposed the project, designed the experiments, and wrote the manuscript; C. J. C. performed the whole experiments; X. P.·Y., Y.·H.·W., R. Z. W., Q. G. Z., M. Q. H., Z. F. Z., H. L. F., J. M., Y.·Y.·H. and J. Y. M. performed the analysis of experimental data; X. F. S. and B. X. H. supervised the whole project.

## Conflicts of interest

There are no conflicts to declare.

## Supplementary Material

SC-012-D1SC02328D-s001
