# Supplementary material for: Quasi-square-shaped cadmium hydroxide nanocatalysts for electrochemical CO2 reduction with high efficiency
Source: Chem Sci. 2021 Aug 10;12(35):11914–20. doi: 10.1039/d1sc02328d (PMC8442700; doi:10.1039/d1sc02328d)
Supplement: SC-012-D1SC02328D-s001 [file SC-012-D1SC02328D-s001.pdf]

Electronic Supplementary Material (ESI) for Chemical Science.
This journal is © The Royal Society of Chemistry 2021

Supporting Information

Quasi-square-shaped Cadmium Hydroxide Nanocatalysts for Electrochemical CO_2 Reduction with High Efficiency

Chunjun Chen,[a,b] Xupeng Yan,[a,b] Ruizhi Wu,[a,b] Yahui Wu,[a,b] Qinggong Zhu,[a] Minqiang Hou,[a] Zhaofu Zhang,[a] Honglei Fan,[a] Jun Ma,[a] Yuying Huang,[e] Jingyuan Ma,[e] Xiaofu Sun,[a,b] * Longfei Lin,[a]* Shoujie Liu,[c,d] * and Buxing Han,[a,b,f] *

[a] Beijing National Laboratory for Molecular Sciences, CAS Key Laboratory of Colloid and Interface and Thermodynamics, CAS Research/Education Center for Excellence in Molecular Sciences
Institute of Chemistry, Chinese Academy of Sciences
Zhongguancun North First Street 2, Beijing, 100190, China
[b] School of Chemistry and Chemical Engineering, University of Chinese Academy of Sciences
Yuquan Road, Shijingshan District, Beijing, 100049, China
[c] Chemistry and Chemical Engineering of Guangdong Laboratory, Shantou 515063, China.
[d] College of Chemistry and Materials Science, Anhui Normal University, Wuhu 241000, China
[e] Shanghai Synchrotron Radiation Facility, Zhangjiang Laboratory (SSRF, ZJLab), Shanghai Advanced Research Institute, Chinese Academy of Sciences, Shanghai 201204, China
[f] Shanghai Key Laboratory of Green Chemistry and Chemical Processes, School of Chemistry and Molecular Engineering, East China Normal University, Shanghai 200062, China

Experimental Section

Materials: $Cd(NO_3)_2 \cdot 4H_2O$, sulfuric acid (95-98%), ethanol (A. R. grade), acetone (A. R. grade), nickel foam, sodium hydroxide (99%) and acetonitrile (A. R. grade, MeCN) were obtained from Sinopharm Chem. Reagent Co. Ltd. sodium citrate (99%), Ag nanoparticles (< 100nm), tannic acid (98%), Nafion N-117 membrane (0.180 mm thick, ≥0.90 meg/g exchange capacity), $AgNO_3$ (99.9%) and poly(tetrafluoroethylene) dispersion liquid (60 wt%/w in water) were purchased from Alfa Aesar China Co., Ltd. 1-Butyl-3-methylimidazolium hexafluorophosphate ([Bmim]PF_6, purity >99%) was obtained from the Centre of Green Chemistry and Catalysis, Lanzhou Institute of Chemical Physics, Chinese Academy of Sciences. N_2 (99.999%) and CO_2 (99.999%) were provided by Beijing Analytical Instrument Company.

Synthetic procedures for Cdhy-QS. In the experiment, 400 mg of $Cd(NO_3)_2 \cdot 4H_2O$ was dispersed in 80 mL of deionized water to form a clear solution by stirring. Subsequently, 160 mg of tannic acid was added, and the solution was stirred to keep a clear solution. Ultimately, 4.0 mL of NaOH (1 mol/L) was added dropwise with continuous stirring under room temperature for 5 min. The final product was collected by centrifuging, washed with deionized water and acetone for three times, and then dried in vacuum overnight. All the experiments mentioned above were conducted at room temperature.

Synthetic procedures for Cdhy-np. The main difference to synthesize Cdhy-np and Cdhy-QS was that the additional order of TA. For Cdhy-np, NaOH and TA were added simultaneously into the solution of $Cd(NO_3)_2$. In the experiment, 400 mg of $Cd(NO_3)_2 \cdot 4H_2O$ was dispersed in 80 mL of deionized water to form a clear solution by stirring. Subsequently, 4.0 mL of NaOH (1 mol/L) with TA (160 mg) was added dropwise, and the solution was stirred for 5 min. The final product was collected by centrifuging, washed with deionized water and acetone for three times, and then dried in vacuum overnight. The experiments were also carried out at room temperature.

Synthetic procedures for Pd-np. The Pd-np was prepared according to the previous literature.[S1] 0.5 mmol $PdCl_2$ (dissolved in 0.1 M HCl solution) and 4 mmol sodium citrate were dissolved into 200 mL deionized water, and then 212.8 mg Vulcan XC-72R carbon black was added, and sonicated for 30 min. 50 mL of 0.1 M $NaBH_4$ solution was added into the suspension dropwise under vigorous stirring at 25 °C. After the suspension was stirred for 8 h, the black precipitate was filtered, washed with water and ethanol, and dried overnight in a vacuum oven at 25 °C.

Materials characterizations: The morphologies of the catalysts were characterized by SEM (HITACHI S-4800) and TEM (JEOL JEM-2100F, equipped with EDS). X-ray photoelectron spectroscopy (XPS) study was performed on the Thermo Scientific ESCALab 250Xi using a 200W Al-Kα radiation. In the analysis chamber, the base

pressure was about 3×10^{-10} mbar. Typically, the hydrocarbon C1s line at 284.8 eV from adventitious carbon was used for energy referencing. The contents of Cd of the catalysts were detected by ICP-AES (Vista-MPX). The X-ray absorption data at the Cd *K*-edge of the samples were recorded at room temperature in transmission mode using ion chambers at beam line BL14W1[S2] of the Shanghai Synchrotron Radiation Facility (SSRF), China. The station was operated with a Si (311) double crystal monochromator. During the measurement, the synchrotron was operated at energy of 3.5 GeV and a current between 150-210 mA. The photon energy was calibrated with the first inflection point of Ag K-edge in Ag foil.

Electrochemical study. All the electrochemical experiments were conducted on the electrochemical workstation (CHI 660E, Shanghai CH Instruments Co., China). Linear sweep voltammetry (LSV) scans were conducted in a single compartment cell, which included a working electrode, a counter electrode (Pt gauzes), and a reference electrode (Ag/Ag^+ with 0.01 M $AgNO_3$ in 0.1 M TBAP-MeCN).[S3] The electrolyte was 0.5 M [Bmim]PF_6-MeCN. The 0.5 M [Bmim]PF_6-MeCN was bubbled with CO_2 or N_2 at least 30 min to ensure the formation of CO_2-saturated or N_2-saturated solution before experiments. LSV measurements were carried out in the potential range of -1.0 V to -2.5 V versus Ag/Ag^+ at a sweep rate of 50 mV s^{-1}.

The electrolysis experiments were performed at 25 °C in a H-type cell,[S4] including a working cathode, a counter anode (platinum gauzes), and a reference electrode (Ag/Ag^+ with 0.01 M $AgNO_3$ in 0.1 M TBAP-MeCN). In the experiments, Nafion-117 membrane was used as proton exchange membrane. In the experiments, the cathode and anode compartments were separated through a Nafion 117 proton exchange membrane. H_2SO_4 aqueous solution (0.5 M) was used as anodic electrolyte. H^+ can be transferred from anode compartment to cathode compartment through Nafion 117 proton exchange membrane, which is the proton source. In each experiment, the amount of anodic/cathodic electrolyte was 30 mL. Before the electrolysis experiment, the catholyte was bubbled with CO_2 for 30 min and the electrolysis was carried out under a steady stream of CO_2 (5 sccm).

Calculation of energy efficiency.

The half-cell EE for the conversion of CO_2 into CO was calculated by the following equation.[S5]

$$EE_{co} = \frac{FE(\%) \times \Delta E_{CO}^{0}}{\Delta E_{CO}}$$

EE_{CO} was the energy efficiency for the conversion of CO_2 into CO. ΔE_{CO}^{0} was the difference between the half reaction potentials for water oxidation (0.6664 V vs Ag/Ag^+) and the reduction of CO_2 into CO (-1.8 V vs Ag/Ag^+). ΔE_{CO} was the difference between the water oxidation potential and the working potential at the cathode,

respectively.

$E_{(Ag/Ag+)} = E_{(RHE)} - 0.54\ V - pH \times 0.059$

The standard half reaction potentials for water oxidation was 1.23 V vs RHE.

EIS study. The EIS measurement was carried out in 0.5 M [Bmim]PF_6-MeCN solution at an open circuit potential (OCP) with an amplitude of 5 mV of 10^{-2} to 10^6 Hz.

Double-layer capacitance (C_{dl}) measurements. The electrochemical active surface area is proportional to C_{dl} value. C_{dl} was determined in H-type electrolysis cell by measuring the capacitive current associated with double-layer charging from the scan-rate dependence of cyclic voltammogram (CV).[S6] The CV ranged from –1.6 V to -1.7 V vs. Ag/Ag^+. The C_{dl} was estimated by plotting the Δj (j_a-j_c) at -1.65 V vs Ag/Ag^+ against the scan rates, in which the j_a and j_c were the anodic and cathodic current density, respectively. The scan rates were 20, 30, 50, 80, 100 and 120 mV s^{-1}.

The ECSA of the working electrodes could be calculated according to the following equation: ECSA = R_fS, where S was the real surface area of the working electrode and R_f was the roughness factor of the working electrode. S was generally equal to the geometric area of working electrode (in this work, S = 1 cm^2). The roughness factor (R_f) can be calculated by the relation $R_f = C_{dl}/a$. The roughness factor of Cdhy-QS was defined to be 1, then the normalized current density can be calculated according to roughness factor of different catalysts.

Computational Method

The free energies of CO_2 reduction states were carried out by the Vienna Ab-initio Simulation Package (VASP),[S7-S8] taking advantage of the DFT with the Projected Augmented Wave (PAW) method.[S9] The exchange and correlation effects were described by the revised Perdew-Burke-Ernzerhof (RPBE) functional.[S10-S11] For all the geometry optimizations, the cutoff energy was set to be 500 eV. The Monkhorst-Pack grids[S12] were set to be 3×3×1, 3×2×1, and 2×2×1 for performing the calculations on (002), (100), and (110) surfaces of Cdhy. A vacuum thickness was added in the z-direction of the simulation box, preventing the interactions between the adjacent slabs.

The reduction of CO_2 to produce CO could occur in the following three elementary steps:

$CO_2 + (H^+ + e^-) + * \rightarrow *COOH$

$*COOH + (H^+ + e^-) \rightarrow *CO + H_2O$

$*CO \rightarrow CO\uparrow + *$

where * denotes the active species on the catalyst surface. Based on the above mechanism, the free energy of two intermediate states, *COOH and *CO, are important to identify the activity of a given material in catalyzing CO_2

reduction. The computational hydrogen electrode (CHE) model[S13] proposed by Nørskov et al. was used to calculate the free energies of CO_2 reduction intermediates, based on which the free energy of an adsorbed species is defined as

$$\Delta G_{ads} = \Delta E_{ads} + \Delta E_{ZPE} - T\Delta S_{ads} + \int C_P dT$$

where ΔE_{ads} is the electronic adsorption energy, ΔE_{ZPE} is the zero-point energy difference between adsorbed and gaseous species, $T\Delta S_{ads}$ is the corresponding entropy difference between these two states, and $\int C_P dT$ is the enthalpy correction. The electronic binding energy is referenced as graphene for each C atom, ½ H_2 for each H atom, and (H_2O-H_2) for each O atom, plus the energy of the clean slab. The corrections of zero-point energy, entropy, and enthalpy of adsorbed and gaseous species can be found in the supplementary information.

The descriptor proposed by Norskov et al. was used to describe the HER activity on a given catalyst surface, where the free energy of hydrogen adsorption (ΔGH*) was considered as the key parameter determining the HER activity.[S14] For HER in aqueous solutions, it is a two-step process and involves only one reaction intermediate, the chemisorbed H atom. The free energy of the adsorbed hydrogen is defined as:

$$\Delta G_{H^*} = \Delta E_H + \Delta E_{ZPE} - T\Delta S_H$$

where ΔE_H is the hydrogen binding energy, ΔE_{ZPE} is the zero point energy difference between adsorbed hydrogen and gaseous hydrogen, and TΔS is the corresponding entropy difference between these two states. According to previous studies, [S15] here we used a 0.24 eV value to represent the correction of zero point energy and entropy of hydrogen state.

Calculation of Faradaic efficiencies of gaseous and liquid products.[S16-S17]

liquid products:

After electrolysis, the ionic liquid in the electrolyte can act as the internal standard. Because the concentration of ionic liquid was known, the moles of liquids products can be calculated from integral areas and calibration curves. The Faradaic efficiency of liquid product is:

$$FE = \frac{moles\ of\ product}{Q/nF} \times 100\%$$

(Q: Charge (C); F：Faradaic constant (96485 C/mol); n: the number of electrons required to generate the product)

Gaseous products:

From the GC peak areas and calibration curves for the TCD detector, we can obtain the V % of gasous products . Since the flow rate of the CO_2 was constent, the moles of gasous products can be calculated. The Faradaic

efficiency of gasous product is:

$$FE = \frac{moles\ of\ product}{Q\ /\ nF} \times 100\%$$

(Q: electric quantity (C); F：Faradaic constant (96485 C/mol); n: the number of electrons required to generate the product)

Supplementary Figures

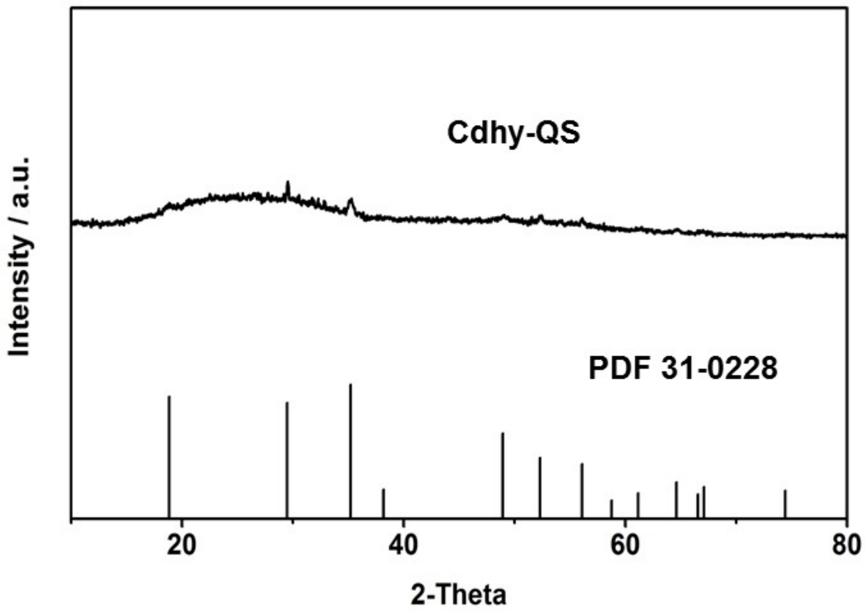

Figure S1. XRD pattern of Cdhy-QS.

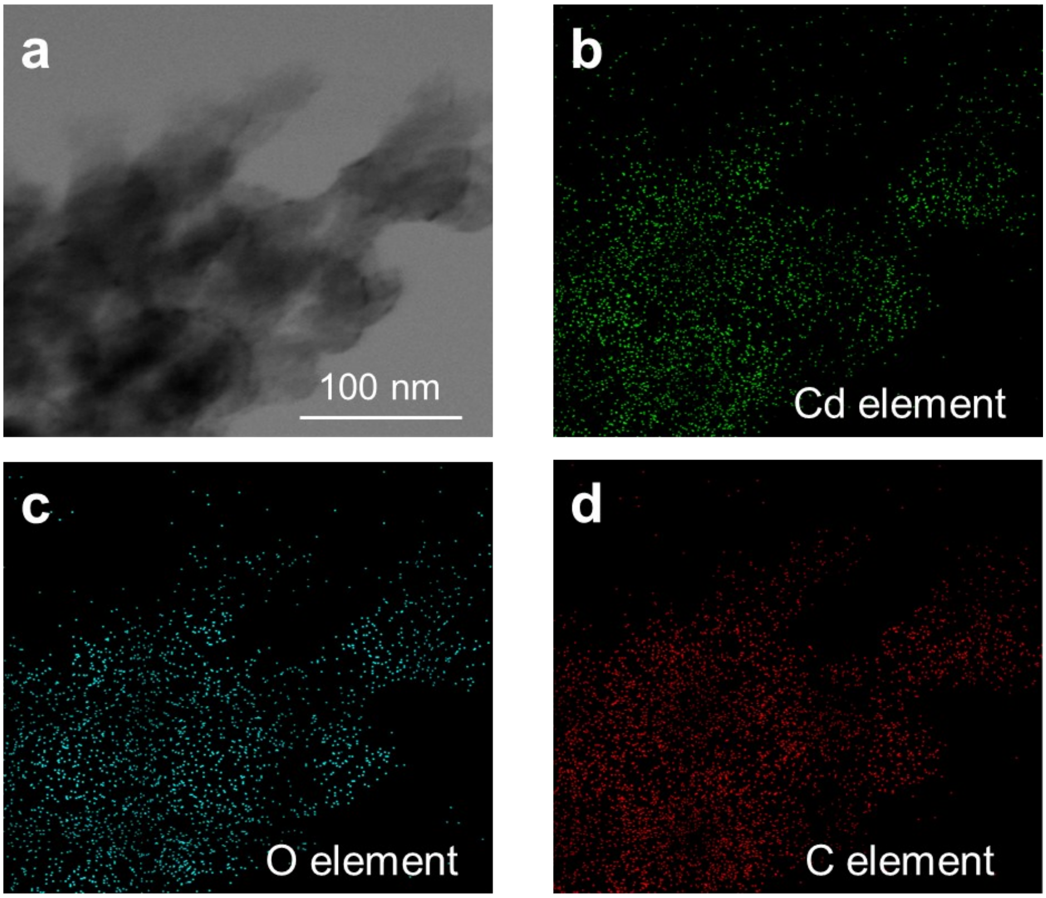

Figure S2. EDS mappings of Cdhy-QS.

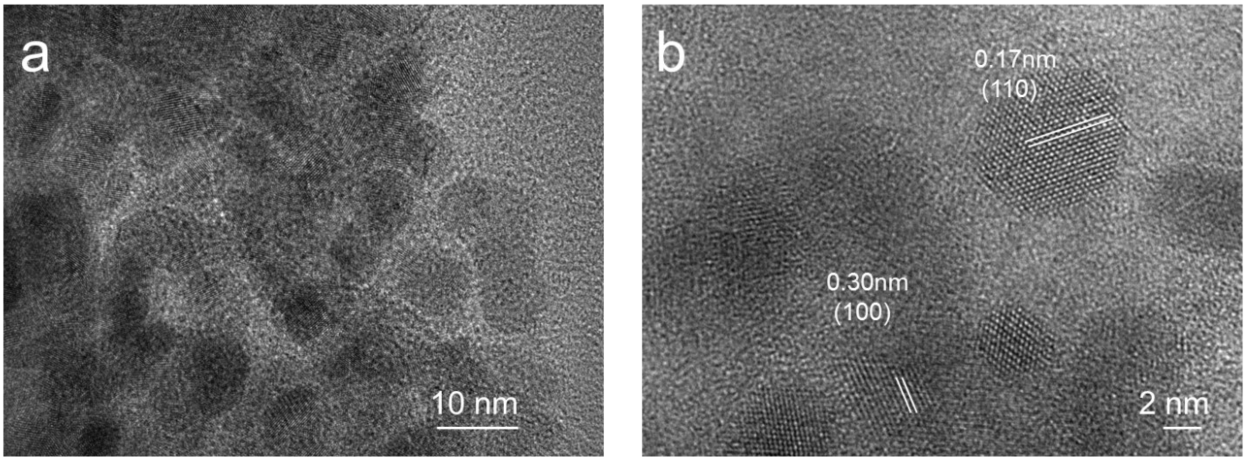

Figure S3. TEM (a) and HRTEM (b) images of Cdhy-np.

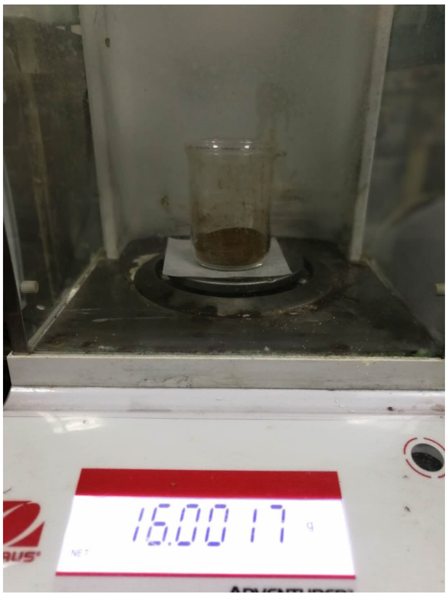

Figure S4. The digital image for the yield of Cdhy-QS.

In order to verify the feasibility of mass production, enlarged experiment was carried out. We obtained about 16 g Cdhy-QS in a 3 L round bottom flask, and more production is limited only by the size of flask in the laboratory.

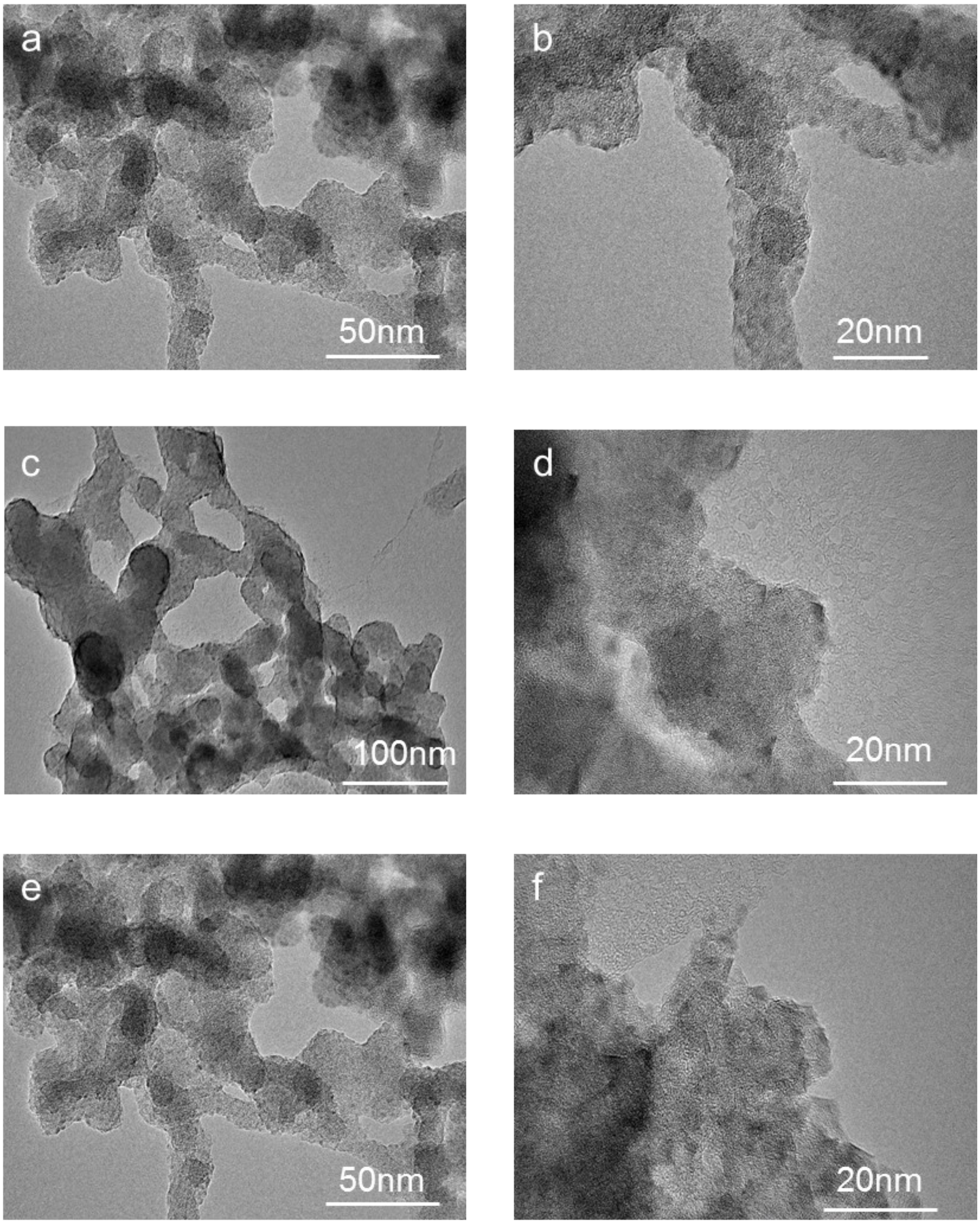

Figure S5. TEM images of Cdhy-QS from different batches. (a, b) the first batch; (c, d) the second batch; (e, f) enlarged experiment to prepare 16 g catalyst in one batch.

In order to verify the feasibility of mass production, repeated experiment was carried out. The difference of the morphologies of the catalysts from different batches were not considerable, indicating the excellent repeatability of the preparation process.

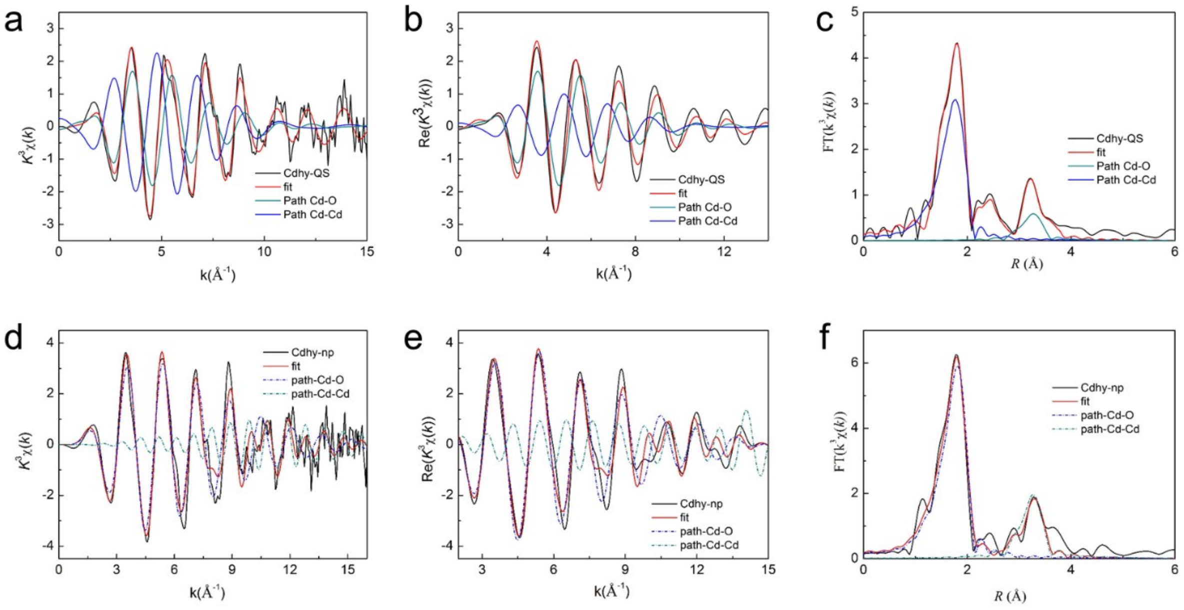

Figure S6. The EXAFS data fitting results of Cdhy-QS and Cdhy-np.

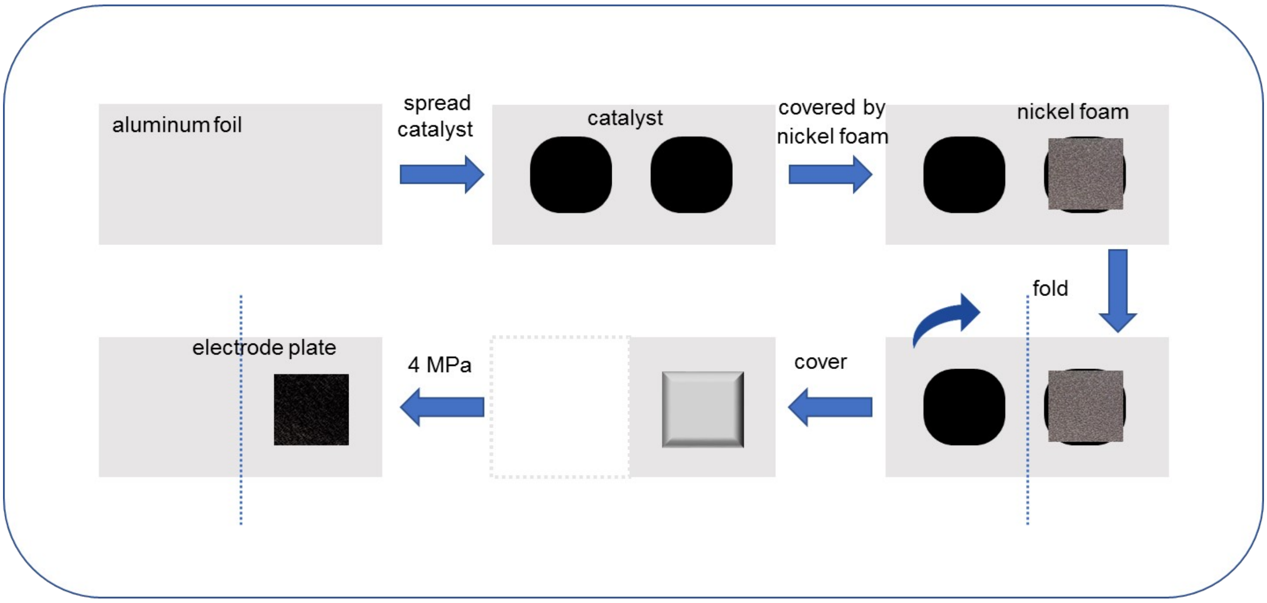

Figure S7. The schematic diagram of preparation process of working electrode.

To prepare the working electrode, the catalysts (2 mg), carbon (XC-72, 1 mg), and poly(tetrafluoroethylene) (10 μl) were dispersed in acetone (1 mL) to form a homogeneous ink. 0.5 mL of the ink was then spread on a clean aluminum foil. Five minutes later, a piece of nickel foam was put on the catalyst, and the nickel foam was covered with another side. Then the mixture and nickel foam were pressed to an electrode plate under 4 MPa by powder pressing machine. The catalyst will be tightly bonded to the nickel foam.

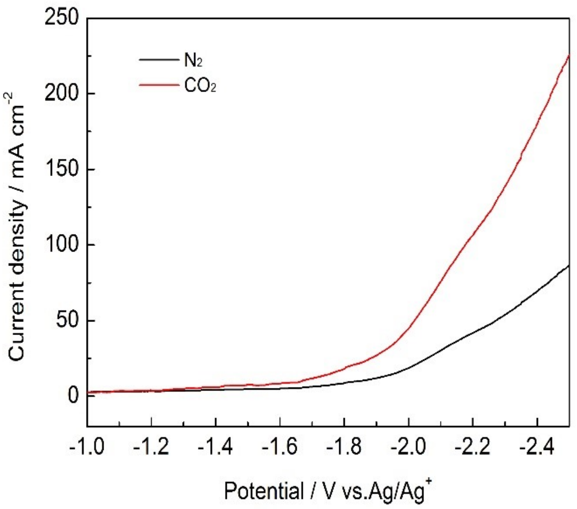

Figure S8. The LSV curves for Cdhy-QS in CO_2 or N_2-saturated 0.5 M [Bmin]PF_6/MeCN electrolyte.

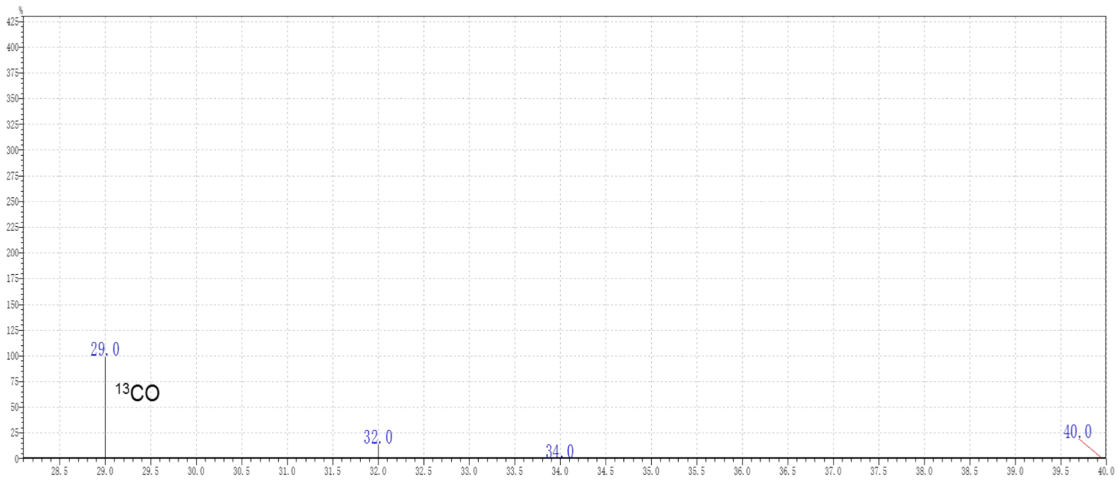

Figure S9. The mass spectrum of ^{13}CO (m/z=29) production from $^{13}CO_2$ over Cdhy-QS.

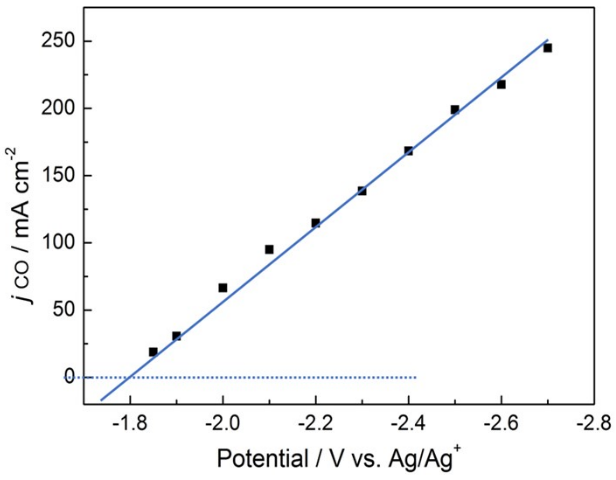

Figure S10. Current densities for CO production under different potentials and the equilibrium potential can be obtained by extrapolation method.

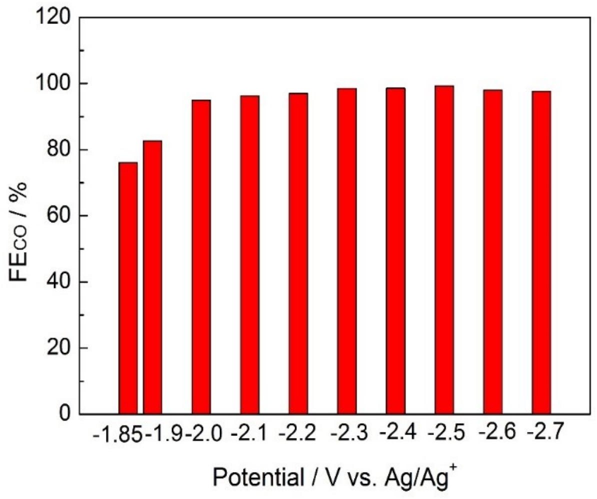

Figure S11. The FE_{CO} for Cdhy-np at different potentials.

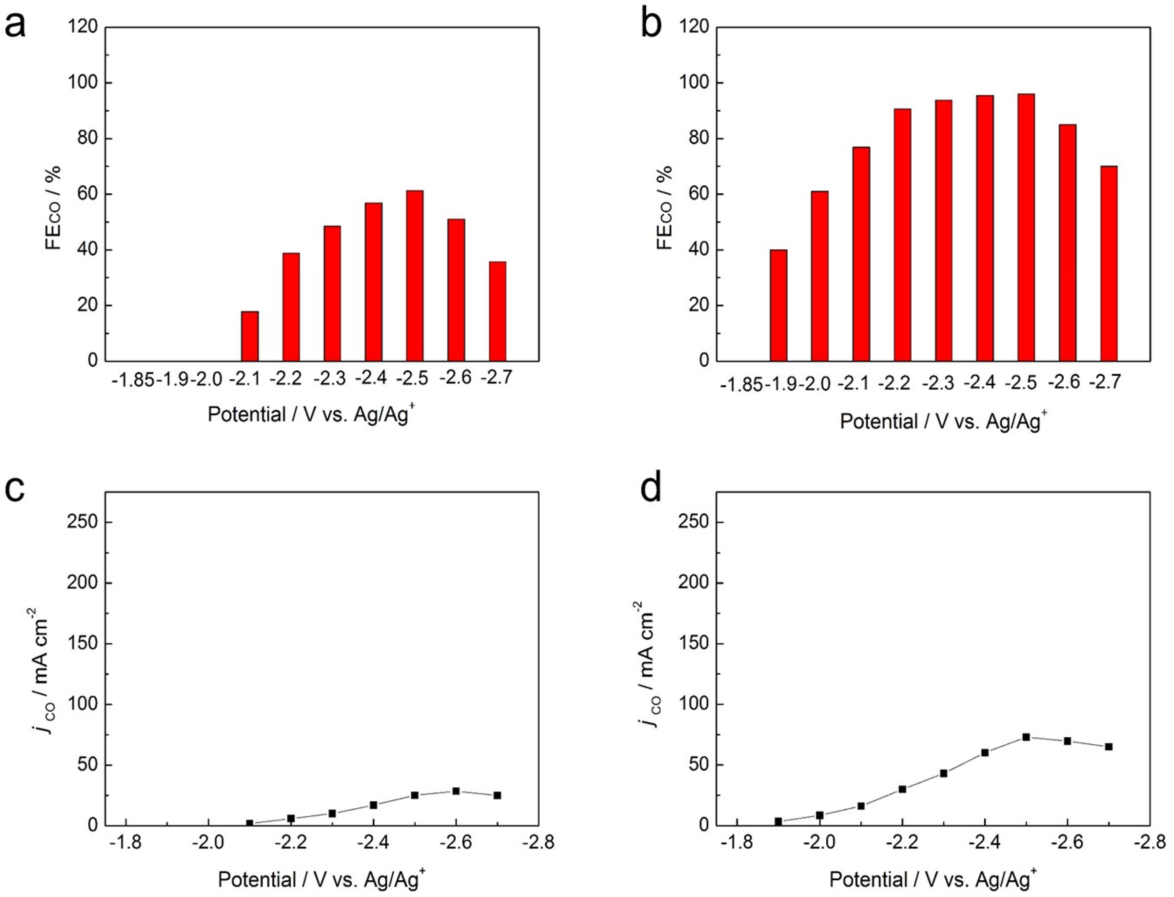

Figure S12. (a) The FE_{CO} for Pd-np at different potentials. (b) The FE_{CO} for Ag-np at different potentials. (c) The current density of CO for Pd-np at different potentials. (d) The current density of CO for Ag-np at different potentials.

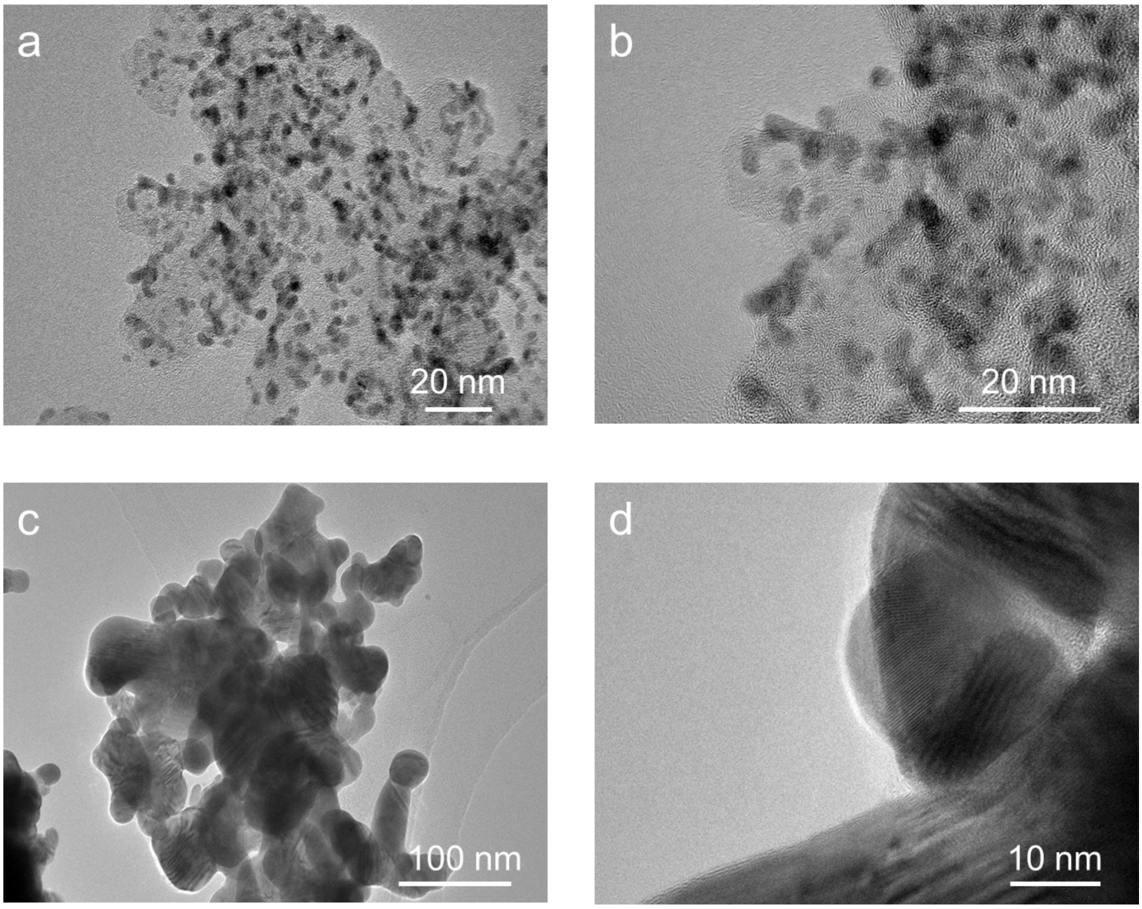

Figure S13. TEM (a) and HR-TEM (b) images of Pd-np. TEM (c) and HR-TEM (d) images of Ag-np.

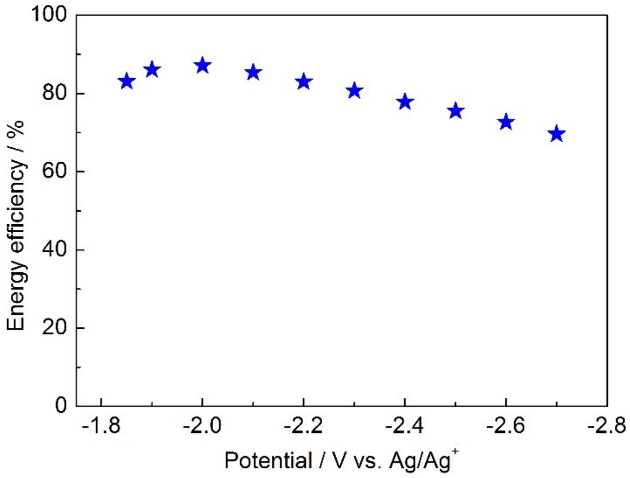

Figure S14. The EE_{CO} for Cdhy-QS at different potentials.

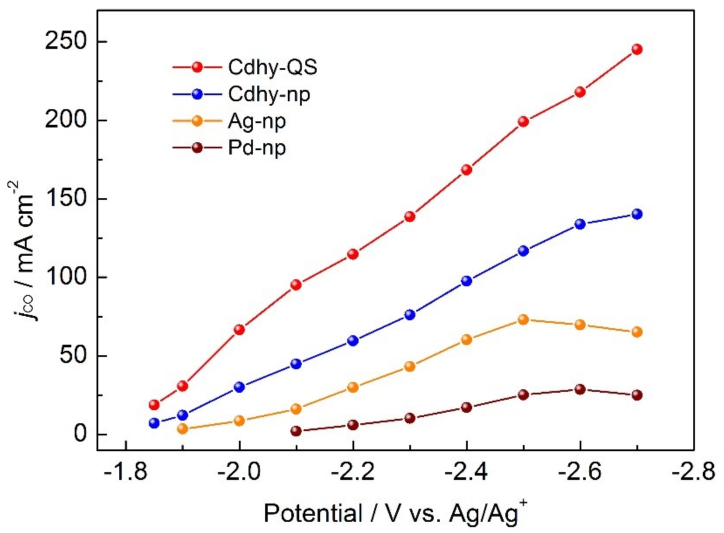

Figure S15. The partial current density for CO production over Cdhy-QS, Cdhy-np, Ag-np and Pd-np.

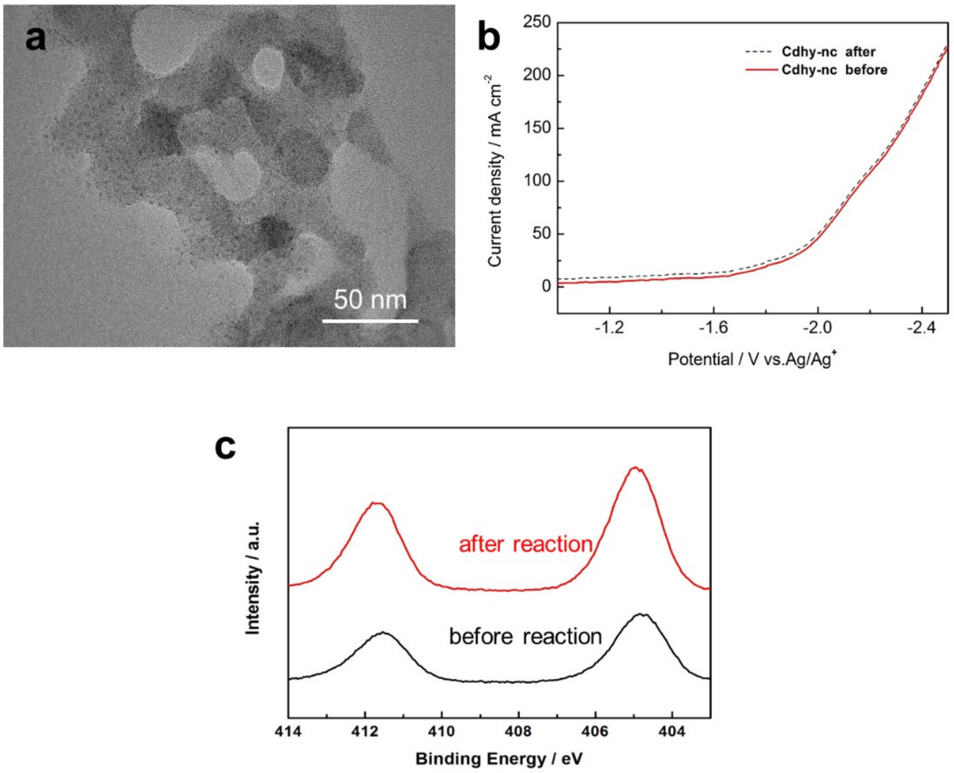

Figure S16. (a) TEM image of Cdhy-QS after CO_2 electroreduction. (b) LSV curves of Cdhy-QS before and after CO_2 electroreduction. (c) The Cd 3d XPS spectra of Cdhy-QS before and after CO_2 electroreduction.

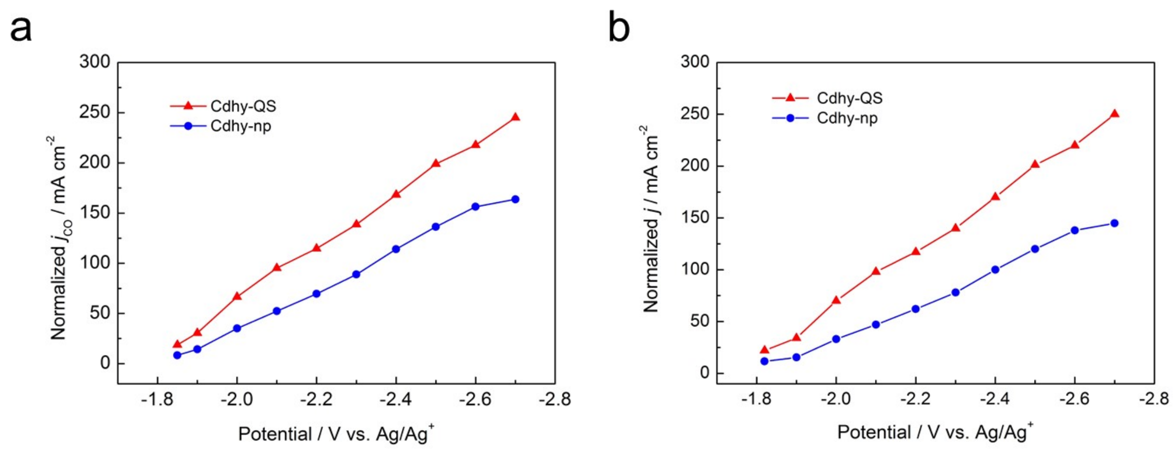

Figure S17. (a) The normalized current density for CO production by ECSA for different catalysts. (b) The normalized total current density by ECSA for different catalysts.

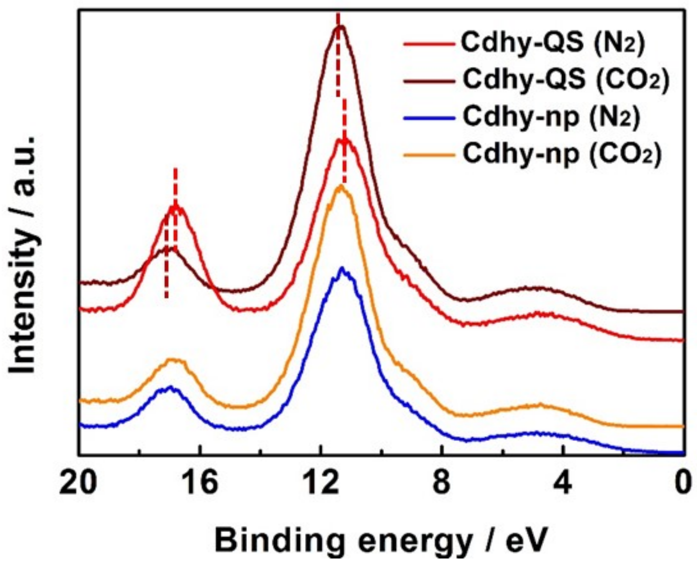

Figure S18. The valence band spectra of Cdhy-QS and Cdhy-np after the electrolysis in different gas atmosphere.

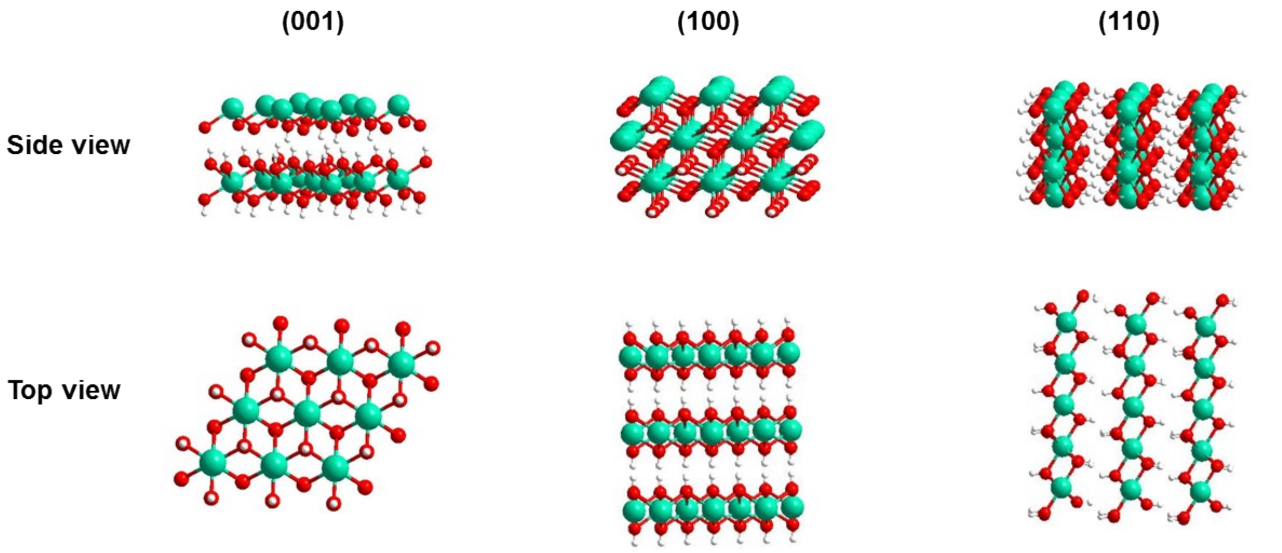

Figure S19. Side view and top view of the (001), (100) and (110) facet of Cdhy. The atoms in cyan, red, and white represent Cd, O, and H, respectively.

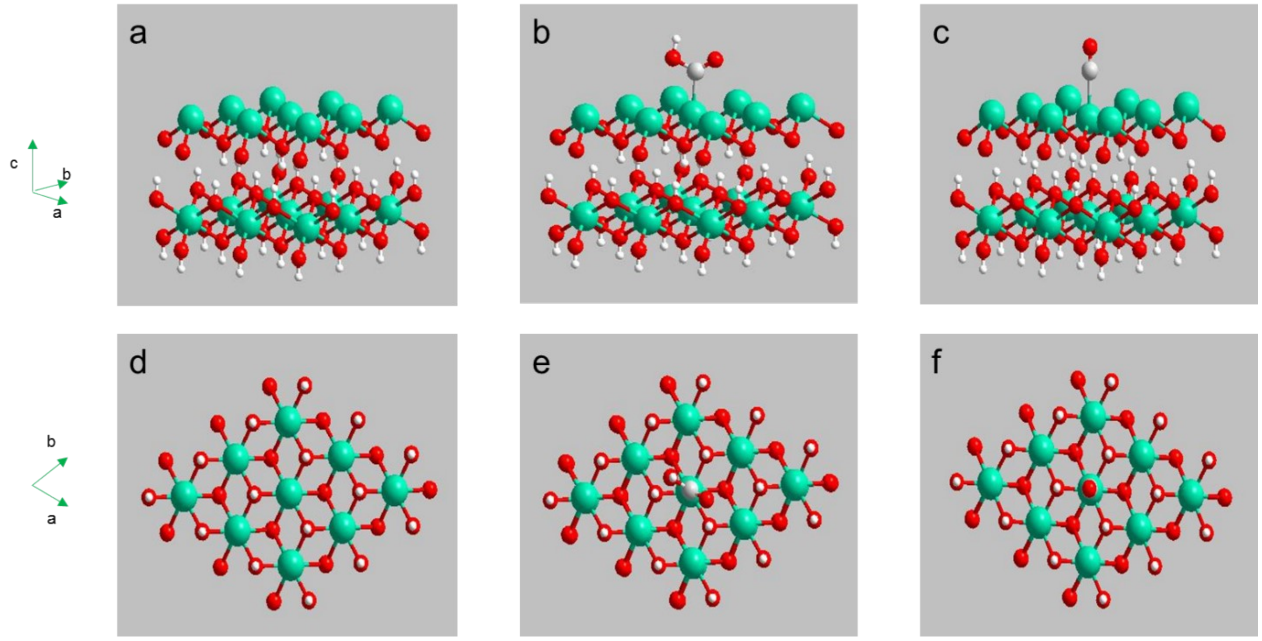

Figure S20. (a) Side view and (d) top view of the (001) facet of Cdhy; (b) Side view and (e) top view of *COOH on the (001) facet of Cdhy; (c) Side view and (f) top view of *CO on the (001) facet of Cdhy. The atoms in cyan, grey, red, and white represent Cd, C, O, and H, respectively.

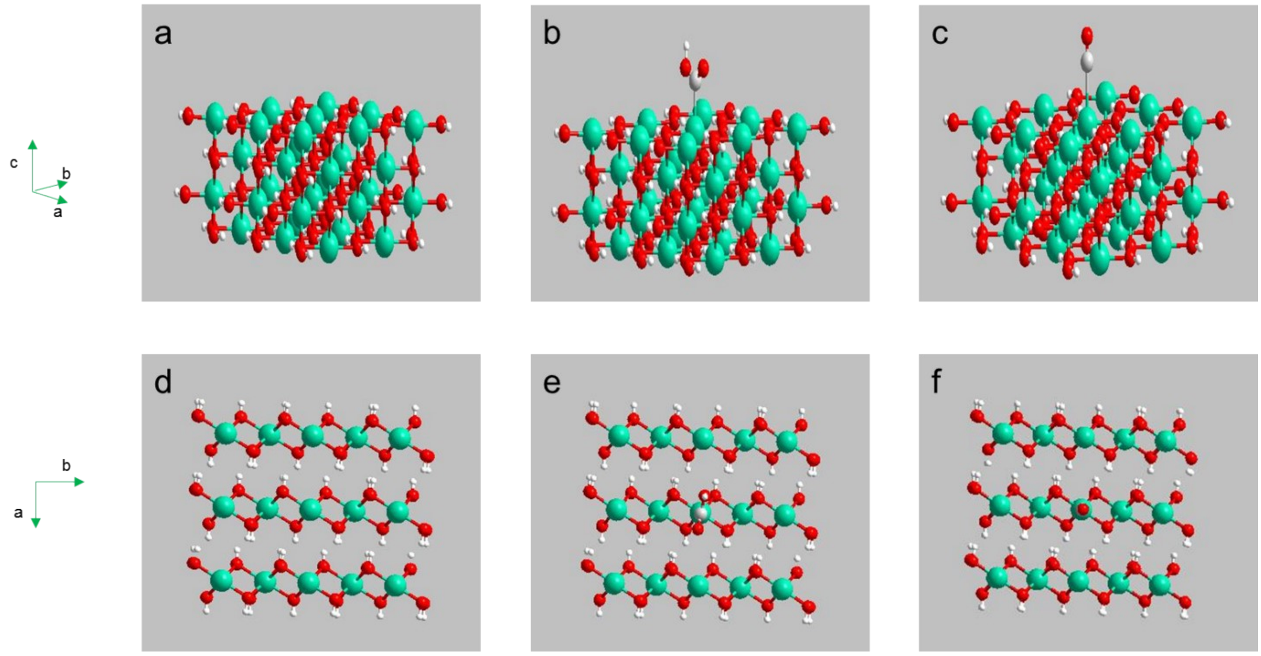

Figure S21. (a) Side view and (d) top view of the (110) facet of Cdhy; (b) Side view and (e) top view of *COOH on the (110) facet of Cdhy; (c) Side view and (f) top view of *CO on the (110) facet of Cdhy. The atoms in cyan, grey, red, and white represent Cd, C, O, and H, respectively.

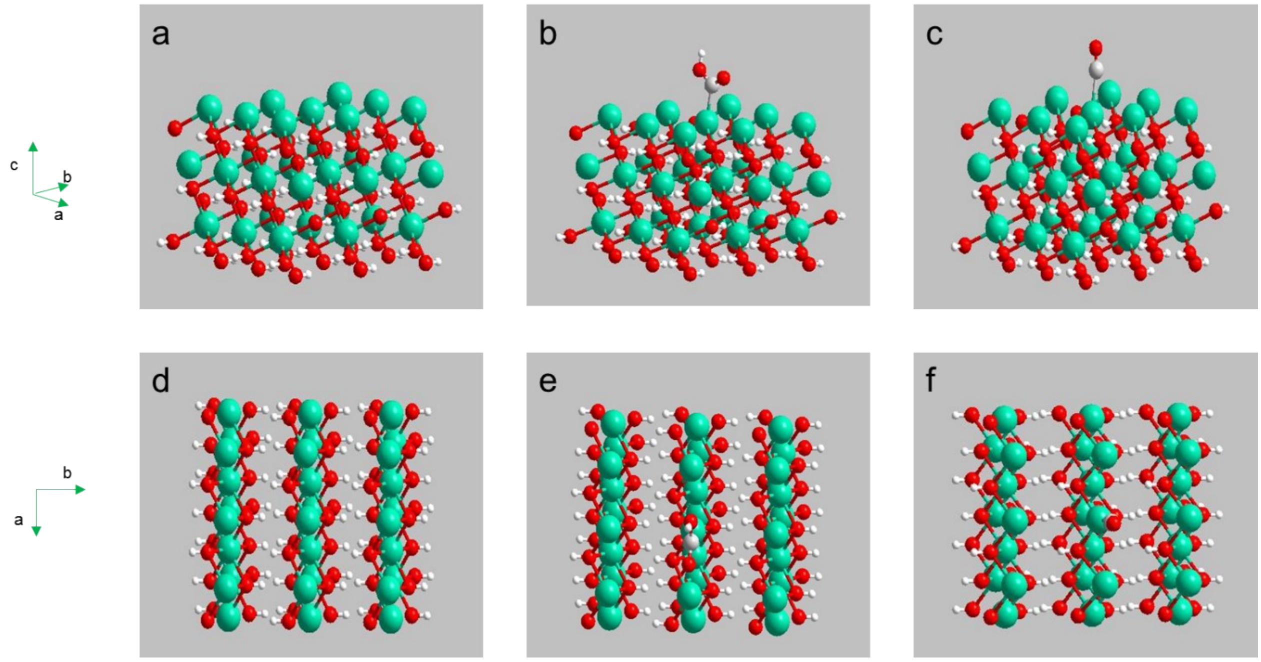

Figure S22. (a) Side view and (d) top view of the (100) facet of Cdhy; (b) Side view and (e) top view of *COOH on the (100) facet of Cdhy; (c) Side view and (f) top view of *CO on the (100) facet of Cdhy. The atoms in cyan, grey, red, and white represent Cd, C, O, and H, respectively.

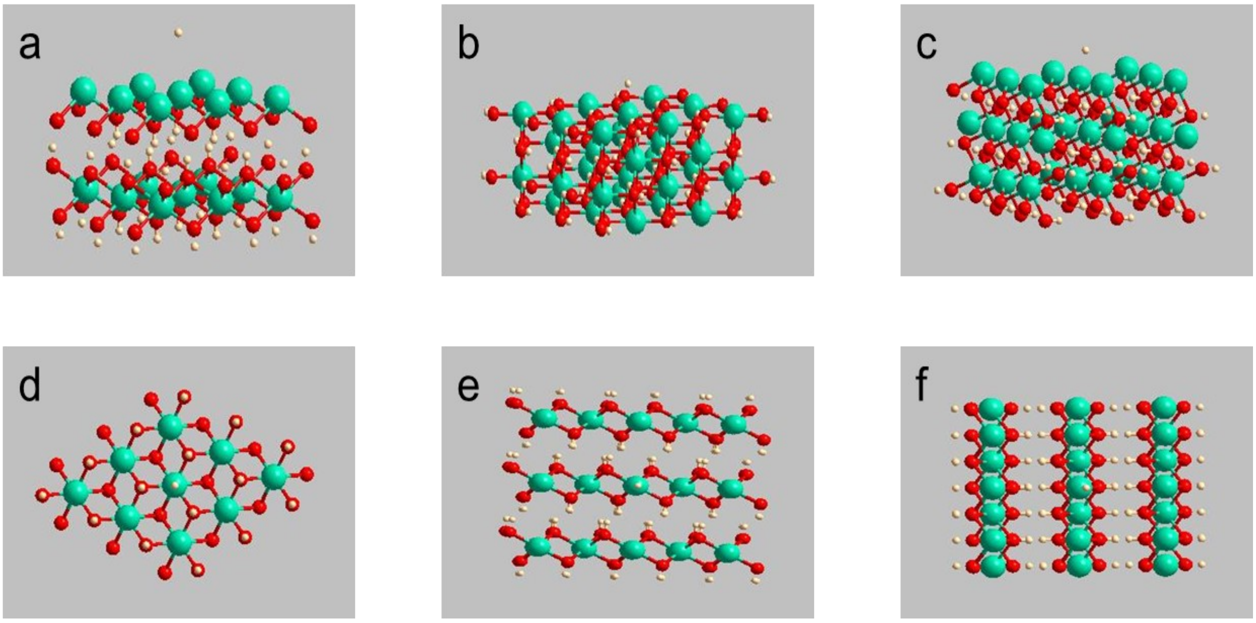

Figure S23. (a) Side view and (d) top view of *H the (002) facet of Cdhy; (b) Side view and (e) top view of *H the (110) facet of Cdhy; (c) Side view and (f) top view of *H the (100) facet of Cdhy. The atoms in cyan, red, and white represent Cd, O, and H, respectively.

Table S1. Structural parameters of Cdhy-QS and Cdhy-np extracted from the EXAFS fitting. (S_0^2=0.80)

Sample	Scattering pair	CN	R(Å)	$\sigma^2 (10^{-3} Å^2)$	ΔE_0(eV)
Cdhy-np	Cd-O	5.7	2.30	6.2±1.5	5.2±1.3
	Cd-Cd	2.2	3.51	7.8±1.4	-3.5±0.9
Cdhy-QS	Cd-O	4	2.31	8.6±0.7	5.3±1.2
	Cd-Cd	1.8	3.46	9.1±0.7	-3.4±0.8

S_0^2 is the amplitude reduction factor; CN is the coordination number; R is interatomic distance (the bond length between central atoms and surrounding coordination atoms); σ^2 is Debye-Waller factor (a measure of thermal and static disorder in absorber-scatterer distances); ΔE_0 is edge-energy shift (the difference between the zero kinetic energy value of the sample and that of the theoretical model). R factor is used to evaluate the goodness of the fitting.

Table S2. The comparison of the reported results for CO_2 electroreduction to CO.

**Catalysts**	**Overpotential (mV)**	**Electrolyte**	**Electrolytic tank**	**FE**	**Current density (mA cm^{-2})**	**reference**
Cdhy-QS	700	0.5 M $[BMIM]PF_6$/ MeCN solution	H-cell	99.5	201.2	This work
	900	0.5 M $[BMIM]PF_6$/ MeCN solution	H-cell	98.1	250.2	This work
Cdhy-np	700	0.5 M $[BMIM]PF_6$/ MeCN solution	H-cell	99.1	116.7	This work
Ag np	700	0.5 M $[BMIM]PF_6$/ MeCN solution	H-cell	96.1	75.9	This work
Pd np	700	0.5 M $[BMIM]PF_6$/ MeCN solution	H-cell	61.3	41.1	This work
WSe_2	650	50 (vol %) $EMIMBF_4$ and 50 (vol %) H_2O	H-cell	90	270	S18
Nb-doped MoS_2	650	50 (vol %) $EMIMBF_4$ and 50 (vol %) H_2O	H-cell	82	240	S19
MoS_2	650	4 (vol %) $EMIMBF_4$ and 96 (vol %) H_2O	H-cell	98	65	S20
Ni/Fe	600	0.5M $KHCO_3$ solution	H-cell	98	7.4	S21
Ag no	400	0.5 M $KHCO_3$ solution	H-cell	90	9	S22

Cu an	500	0.1M $KHCO_3$ solution	H-cell	33	2.8	S23
Cu pc	700	0.1M $KHCO_3$ solution	H-cell	20	0.9	S23
Au pc	280	0.5 M $NaHCO_3$,	H-cell	40	0.5	S24
unsaturated nickel-nitrogen	700	1 M $KHCO_3$ solution	H-cell	95	60	S25
N, S codoped carbon nanofiber	600	0.1 M $KHCO_3$ solution	H-cell	94	103	S26
Isolated Ni single atoms in graphene nanosheets	600	0.5 M $KHCO_3$ solution	H-cell	98	12	S27
Nickel Single-Atom Catalysts	900	0.5 M $KHCO_3$ solution	H-cell	98	18	S28
Single-Atom $Co-N_5$ catalyst	700	0.2 M $KHCO_3$ solution	H-cell	99	5	S29
Core/Shell Cu/SnO_2	700	0.5 M $KHCO_3$ solution	H-cell	89	15	S30
Copper-Indium Bimetallic	600	0.1 M $KHCO_3$ solution	H-cell	92	17	S31
Ultrathin Palladium Nanosheets	700	0.1 M $KHCO_3$ solution	H-cell	92	11	S32
CoPc/CNT	500	0.1 M $KHCO_3$	H-cell	91	8.7	S33

		solution				
Tri-Ag-NPs	700	0.1 M $KHCO_3$ solution	H-cell	96.8	1.2	S34
Pd NP	700	0.1 M $KHCO_3$ solution	H-cell	90	7	S1
Vo-rich ZnO nanosheets	1000	0.1 M $KHCO_3$ solution	H-cell	83	16	S35

Table S3. The results of C_{dl} for Cdhy-QS and Cdhy-np.

**Catalysts**	**The slope (mF cm^{-2})**	**C_{dl} (mF cm^{-2})**
Cdhy-QS	37	18.5
Cdhy-np	32	16

Table S4. The correction of zero point energy, enthalpy effect and entropy effect of the adsorbed and gaseous species

	ZPE(eV)	$\int C_P dT$(eV)	TS(eV)
*COOH	0.62	0.10	0.18
*CO	0.19	0.08	0.15
CO_2	0.31	0.10	0.65
H_2O	0.58	0.10	0.65
H_2	0.27	0.09	0.42

Table S5. The electroreduction CO_2 activity over o-Cdhy using the low content CO_2.

The content of CO_2 (mol %)	Current density (mA cm^{-2})	Faradic efficiency (%)	The content of products (mol %)		
			CO	H_2	CO_2
4.9	143	84.6	37.1	6.4	56.5
10.1	161	85.9	49.3	5.5	45.2
14.8	173	94.4	57.2	3.1	39.7

References

S1 D. Gao, H. Zhou, J. Wang, S. Miao, F. Yang, G. Wang, J. Wang, X. Bao, *J. Am. Chem. Soc.* **2015**, *137*, 4288-4291.

S2 Yu, H-S. *et al.* The XAFS beamline of SSRF. H. -S. Yu, X. -J. Wei, J. Li, S. -Q. Gu, S. Zhang, L. -H. Wang, J. -Y. Ma, L. -N. Li, Q. Gao, R. Si, F. -F. Sun, Y. Wang, F. Song, H. -J. Xu, X. -H. Yu, Y. Zou, J. -Q. Wang, Z. Jiang, Y. -Y. Huang, *Nucl. Sci. Tech.* **2015**, 26, 050102.

S3 Q. Zhu, J. Ma, X. Kang, X. Sun, H. Liu, J. Hu, Z. Liu, B. Han, *Angew. Chem. Int. Ed.* **2016**, *55*, 9012-9016.

S4 X. Sun, Q. Zhu, X. Kang, H. Liu, Q. Qian, Z. Zhang, B. Han, *Angew. Chem. Int. Ed.* **2016**, *55*, 6771-6775.

S5 S. Peng, F. Gong, L. Li, D. Yu, D. Ji, T. Zhang, Z. Hu, Z. Zhang, S. Chou, Y. Du, S. Ramakrishna, *J. Am. Chem. Soc.* **2018**, *140*, 13644-13653.

S6 S. Gao, Y. Lin, X. Jiao, Y. Sun, Q. Luo, W. Zhang, D. Li, J. Yang, Y. Xie, *Nature* **2016**, *529*, 68-71.

S7 G. Kresse, J. Furthmüller, *Phys. Rev. B,* **1996**, *54*, 11169-11186.

S8 Kresse, G. & Hafner, J. Ab initio. *Phys. Rev. B* **49**, 14251-14269 (1994).

S9 P. E. Blöchl, *Phys. Rev. B*, **1994,** *50*, 17953-17979.

S10 J. P. Perdew, K. Burke, and M. Ernzerhof, *Phys. Rev. Lett.* **1996**, *77*, 3865-3868.

S11 Y. Zhang, W. Yang, *Phys. Rev. Lett.* **1998**, *80*, 890-890.

S12 B. Hammer, L. B. Hansen and J. K. Nørskov, *Phys. Rev. B,* **1999**, *59*, 7413-7421.

S13 H. J. Monkhorst, J. D. Pack, *Phys. Rev. B*, **1976**, *13*, 5188.

S14 J. K. Nørskov, J. Rossmeisl, A. Logadottir and L. Lindqvist, *J. Phys. Chem. B*, **2004**, *108*, 17886-17892.

S15 Nørskov, J. K. *et al.* Trends in the exchange current for hydrogen evolution. *J. Electrochem. Soc.*, **152**, J23-J26 (2005).

S16 Chen, C. *et al.* Efficient electroreduction of CO_2 to C2 products over B-doped Oxide-derived copper. *Green Chem.*, **20**, 4579-4583 (2018).

S17 X. Sun, C. Chen, S. Liu, S. Hong, Q. Zhu, Q. Qian, B. Han, J. Zhang, L. Zheng, *Angew. Chem. Int. Ed.* **2019**, *58*, 4669-4673.

S18 M. Asadi, K. Kim, C. Liu, A. V. Addepalli, P. Abbasi, P. Yasaei, P. Phillips, A. Behranginia, J. M. Cerrato, R. Haasch, P. Zapol, B. Kumar, R. F. Klie, J. Abiade, L. A. Curtiss, A. Salehi-Khojin, *Science* **2016**, *353*, 467-470.

S19 P. Abbasi, M. Asadi, C. Liu, S. Sharifi-Asl, B. Sayahpour, A. Behranginia, P. Zapol, R. Shahbazian-Yassar, L. A. Curtiss, A. Salehi-Khojin, *ACS Nano* **2017**, *11*, 453-460.

S20 Asadi, M. *et al.* Robust carbon dioxide reduction on molybdenum disulphide edges. *Nat. Commun.* **5**, 4470 (2014).

S21 W. Ren, X. Tan, W. Yang, C. Jia, S. Xu, K. Wang, S. C. Smith, C. Zhao, *Angew. Chem. Int. Ed.* **2019**, *58*, 6972-6976.

S22 Lu, Q. *et al.* A selective and efficient electrocatalyst for carbon dioxide reduction. *Nat. Commun.* **5**, 4242 (2014).

S23 C. W. Li, M. W. Kanan, *J. Am. Chem. Soc.* **2012**, *134*, 7231-7234.

S24 Y. Chen, C. W. Li, M. W. Kanan, *J. Am. Chem. Soc.* **2012**, *134*, 19969-19972.

S25 C. Yan, H. Li, Y. Ye, H. Wu, F. Cai, R. Si, J. Xiao, S. Miao, S. Xie, F. Yang, Y. Li, G. Wang, X. Bao, *Energy Environ. Sci.* **2018**, *11*, 1204-1210.

S26 H. Yang, Y. Wu, Q. Lin, L. Fan, X. Chai, Q. Zhang, J. Liu, C. He, Z. Lin, *Angew. Chem. Int. Ed.* **2018**, *57*, 15476-15480.

S27 K. Jiang, S. Siahrostami, T. Zheng, Y. Hu, S. Hwang, E. Stavitski, Y. Peng, J. Dynes, M. Gangisetty, D. Su, K. Attenkofer, H. Wang, *Energy Environ. Sci.* **2018**, *11*, 893-903.

S28 Yang, J. *et al.* In Situ Thermal Atomization To Convert Supported Nickel Nanoparticles into Surface-Bound Nickel Single-Atom Catalysts. *Angew. Chem. Int. Ed.* **57**, 14095-14100 (2018).

S29 Y. Pan, R. Lin, Y. Chen, S. Liu, W. Zhu, X. Cao, W. Chen, K. Wu, W. C. Cheong, Y. Wang, L. Zheng, J. Luo, Y. Lin, Y. Liu, C. Liu, J. Li, Q. Lu, X. Chen, D. Wang, Q. Peng, C. Chen, Y. Li, *J. Am. Chem. Soc.* **2018**, *140*, 4218-4221.

S30 Q. Li, J. Fu, W. Zhu, Z. Chen, B. Shen, L. Wu, Z. Xi, T. Wang, G. Lu, J.-j. Zhu, S. Sun, *J. Am. Chem. Soc.* **2017**, *139*, 4290-4293.

S31 S. Rasul, D. H. Anjum, A. Jedidi, Y. Minenkov, L. Cavallo, K. Takanabe, *Angew. Chem. Int. Ed.* **2015**, *54*, 2146-2150.

S32 W. Zhu, L. Zhang, P. Yang, C. Hu, Z. Luo, X. Chang, Z. J. Zhao, J. Gong, *Angew. Chem. Int. Ed.* **2018**, *57*, 11544-11548.

S33 X. Zhang, Z. Wu, X. Zhang, L. Li, Y. Li, H. Xu, X. Li, X. Yu, Z. Zhang, Y. Liang, H. Wang, *Nat. Commun.* **2017**, *8*, 14675.

S34 S. Liu, H. Tao, L. Zeng, Q. Liu, Z. Xu, Q. Liu, J.-L. Luo, *J. Am. Chem. Soc.* **2017**, *139*, 2160-2163.

S35 Z. Geng, X. Kong, W. Chen, H. Su, Y. Liu, F. Cai, G. Wang, J. Zeng, *Angew. Chem. Int. Ed.* **2018**, *57*, 6054-6059.
